# L-type Amino Acid Transporter 1 Utilizing Ferulic Acid Derivatives Show Increased Drug Delivery in the Mouse Pancreas Along with Decreased Lipid Peroxidation and Prostaglandin Production

**DOI:** 10.1021/acs.molpharmaceut.2c00328

**Published:** 2022-08-26

**Authors:** Janne Tampio, Magdalena Markowicz-Piasecka, Ahmed Montaser, Jaana Rysä, Anu Kauppinen, Kristiina M. Huttunen

**Affiliations:** † School of Pharmacy, Faculty of Health Sciences, University of Eastern Finland, P.O. Box 1627, FI-70211Kuopio, Finland; ‡ Laboratory of Bioanalysis, Department of Pharmaceutical Chemistry, Drug Analysis and Radiopharmacy, Medical University of Lodz, ul. Muszyńskiego 1, 90-151Lodz, Poland

**Keywords:** hemocompatibility, L-type amino acid transporter 1 (LAT1), oxidative stress, pharmacokinetics, pharmacoproteomics, transporter-mediated drug delivery

## Abstract

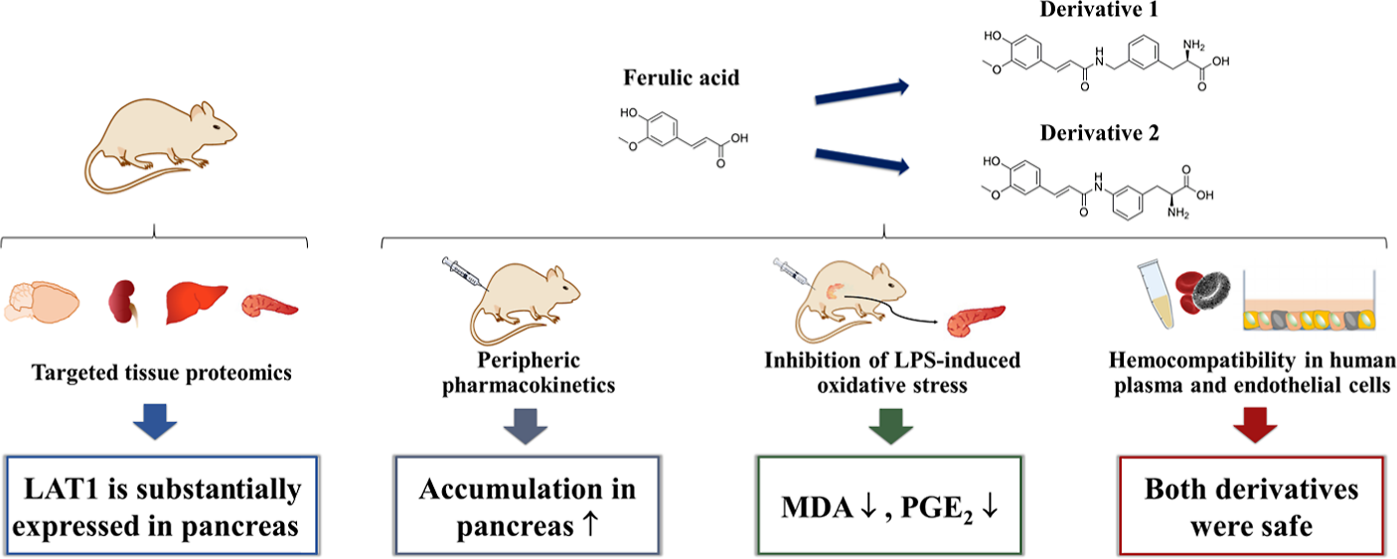

Oxidative stress and pathological changes of Alzheimer’s disease (AD) overlap with metabolic diseases, such as diabetes mellitus (DM). Therefore, tackling oxidative stress with antioxidants is a compelling drug target against multiple chronic diseases simultaneously. Ferulic acid (FA), a natural antioxidant, has previously been studied as a therapeutic agent against both AD and DM. However, FA suffers from poor bioavailability and delivery. As a solution, we have previously reported about L-type amino acid transporter 1 (LAT1)-utilizing derivatives with increased brain delivery and efficacy. In the present study, we evaluated the pharmacokinetics and antioxidative efficacy of the two derivatives in peripheral mouse tissues. Furthermore, we quantified the LAT1 expression in studied tissues with a targeted proteomics method to verify the transporter expression in mouse tissues. Additionally, the safety of the derivatives was assessed by exploring their effects on hemostasis in human plasma, erythrocytes, and endothelial cells. We found that both derivatives accumulated substantially in the pancreas, with over a 100-times higher area under curve compared to the FA. Supporting the pharmacokinetics, the LAT1 was highly expressed in the mouse pancreas. Treating mice with the LAT1-utilizing derivative of FA lowered malondialdehyde and prostaglandin E_2_ production in the pancreas, highlighting its antioxidative efficacy. Additionally, the LAT1-utilizing derivatives were found to be hemocompatible in human plasma and endothelial cells. Since antioxidative derivative 1 was substantially delivered into the pancreas along the previously studied brain, the derivative can be considered as a safe dual-targeting drug candidate in both the pancreas and the brain.

## 1 Introduction

Diabetes mellitus (DM) is a group of metabolic diseases characterized by chronic hyperglycemia. Hyperglycemia disturbs cell homeostasis in many ways and induces production of reactive oxygen species (ROS).^1^ The increased amount of ROS causes oxidative stress, which induces low-level chronic inflammation and further impairs the glucose uptake in adipocytes and skeletal muscles.^2,3^ Chronic inflammation can lead to severe comorbidities, such as diabetic neuro- and nephropathy,^1^ and predispose to neurodegenerative diseases, such as Alzheimer’s (AD) and Parkinson’s disease (PD), which also share mutual pathological changes with DM.^4,5^ As the number of DM patients is growing worldwide, attenuating oxidative stress is a compelling drug target to prevent associated diseases.^6^

One promising drug candidate against oxidative stress is ferulic acid (FA) (4-hydroxy-3-methoxycinnamic acid), which is a ubiquitous phenolic acid present in various plants, such as grains, coffee seeds, and nuts.^7,8^ It has shown beneficial effects in study models of chronic diseases. For example, FA lowered blood glucose levels, ROS production, and reverted diabetes-induced spleen damage caused by streptozotocin induction in rats with a daily 50 mg/kg oral dose.^9^ Additionally, it improved kidney functionality in obese diabetic rats with a daily 10 mg/kg oral dosage.^10^ Moreover, it also decreased the aggregation of the amyloid β (Aβ) peptide in human lens epithelial cells^11^ and to some extent also in mouse brain when administered in tap water.^12^ Aβ aggregation has been associated with increased ROS production, promoting oxidative stress as an intervention target.^13,14^ In addition to the pathological changes, FA has also alleviated motoric symptoms of PD in a rotarod test with mice after exposure to MPTP (1-methyl-4-phenyl-1,2,3,6- tetrahydropyridine),^15^ suggesting beneficial effects against PD as well. While all these findings support the use of FA as a therapeutic agent, it suffers from poor bioavailability.^16^

**Figure 1:**
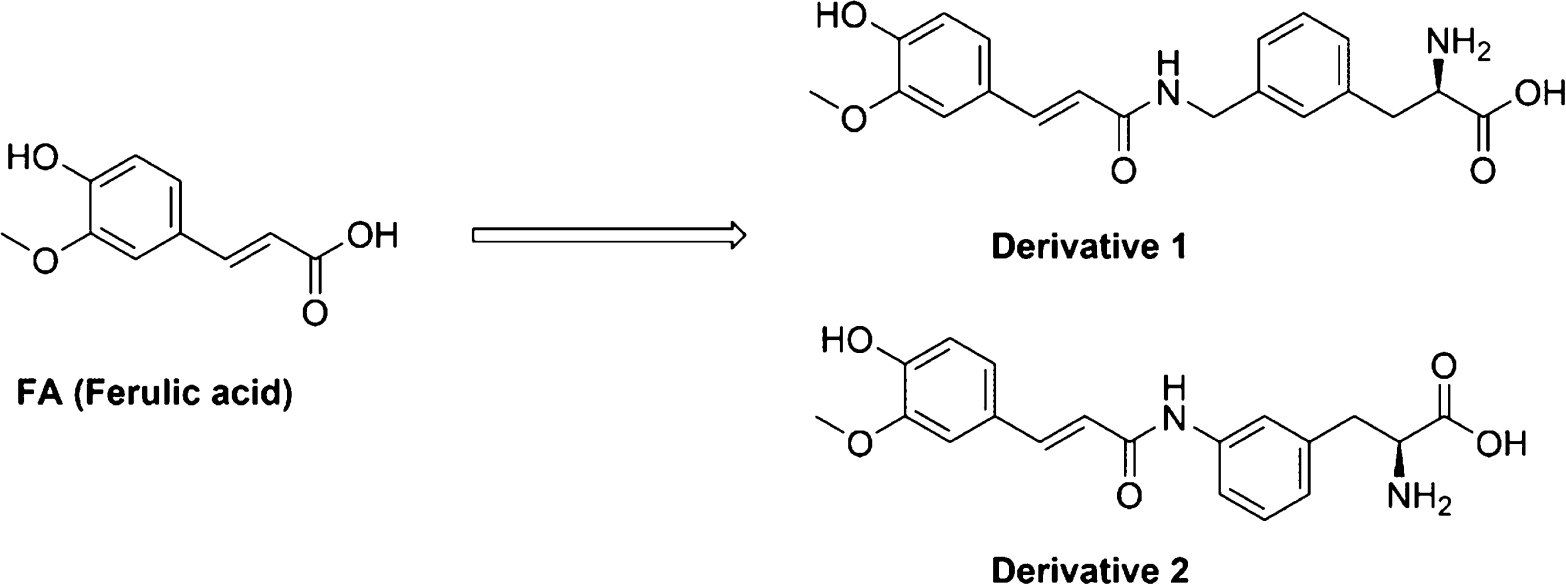
Chemical structures of FA and its LAT1-utilizing derivatives 1 and 2 (D1 and D2).

We have previously reported that amino acid derivatives of FA have increased delivery across the blood–brain barrier (BBB) as well as cellular uptake into neurons and glial cells compared to FA.^17,18^ These derivatives can also alleviate oxidative stress in astrocytes, as lipid peroxidation was significantly reduced after treatment *in vitro*.^18^ Derivatives 1 and 2 (D1 and D2) (Figure 1) have been designed to utilize an L-type amino acid transporter 1 (LAT1), which is a solute carrier protein consisting of two dimers, a light chain (LAT1; *SLC7A5*) and a heavy chain (4F2hc; *SLC3A2*).^19^ It transports not only large, neutral, aromatic, and branched L-configured amino acids but also drugs with appropriate structures, such as levodopa and pregabalin.^20,21^ Therefore, LAT1 is a compelling target to increase drug delivery.

One feasible LAT1 target is the pancreas. There, natural amino acids transported via LAT1 have a significant role in normal pancreatic β-cell functions, such as insulin secretion and regulation of the mammalian target of rapamycin complex 1.^22,23^ We have previously reported notable accumulation of other LAT1-utilizing prodrugs in the pancreas.^24^ Thus, in this study, we evaluated the LAT1-associated pharmacoproteomics in the pancreas of mice. Moreover, the effects of derivatives on human plasma hemostasis and blood circulation system were evaluated, along with preliminary *in vivo* efficacy in the peripheric tissues. Overall, these findings help us to understand the distribution of LAT1 derivatives more widely and to design novel (pro)drugs utilizing the LAT1.

## 2 Experimental Section

### 2.1 Chemicals and Materials

All reagents and solvents used in analytical studies were commercial with high purity of analytical grade or ultrapure high-performance liquid chromatography (HPLC) grade. The used water was purified using a Milli-Q Gradient system (Millipore, Milford, MA, USA). The studied LAT1-utilizing derivatives (D1 and D2) of FA were synthesized in-house. Their structural characterization with a ^1^H and ^13^C nuclear magnetic resonance and an electrospray ionization mass spectrometer (ESI-MS), as well as purity (elemental analysis, over 95%), have been confirmed in an earlier publication.^17^ The preparation of human plasma and red blood cells (RBCs) for the coagulation and erythrotoxicity assays was conducted as described previously.^25^

### 2.2 Ethical Issues

The hemocompatibility studies using biological materials were approved by the Bioethics Committee of the Medical University of Lodz (RNN/109/16/KE). The experimental procedures involving mice were made in compliance with the European Commission directives 2010/63/EU and 86/609 and were approved by the Institutional Animal Care and Use Committee of the University of Eastern Finland (Animal Usage Plan number ESAVI/3347/04.10.07/2015). All efforts were made to minimize the number of animals used and to minimize their suffering.

### 2.3 Animals

Healthy 8-week-old male mice (C57BL/6JOlaHsd) weighing 30 ± 5 g were supplied by Envigo (Venray, Netherlands). Mice were housed in stainless steel cages on the 12 h light (7:00 to 19:00) and 12 h dark (19:00–7:00) cycles at the ambient temperature of 22 ± 1 °C with the relative humidity of 50–60%. Tap water and food pellets (Lactamin R36; Lactamin AB, Södertälje, Sweden) were available *ad libitum*. The experiments were carried out during the light phase.

### 2.4 Quantitation of Membrane Transporter Expression *In Vivo*

Both light and heavy chains of the functional LAT1 dimer protein LAT1 and 4F2hc, respectively, were quantified from the crude membrane fraction of mouse brain, kidney, liver, and pancreas homogenates (*n* = 3 per studied tissue type, each from a separate mouse) by a targeted proteomic approach using a triple quadrupole mass spectrometer (QQQ 6495, Agilent Technologies, Santa Clara, CA, USA). To enhance the comparison of protein levels between tissues, glucose transporter 1 (GLUT1) and the alpha subunits 1–3 of the Na^+^/K^+^-ATPase were quantified simultaneously. Briefly, the crude membrane fractions were isolated from homogenized tissues using a commercial membrane protein extraction kit (BioVision, Inc., Milpitas, CA, USA) by following the manufacturer’s protocol. The marker peptides specific for each protein were selected with an *in silico* protocol described^26^ and experimentally validated^27^ earlier. The selected marker peptide sequences were purchased from JPT company (JPT Peptide Technologies GmbH, Berlin, Germany) as isotopically labeled and absolutely quantified tryptic peptides. After trypsin digestion, samples containing the native and isotopically labeled peptides were quantified using 2–3 transitions of precursor and product ions using multiple reaction monitoring (MRM) mode. The protein concentrations were quantified based on the ratio between the native digested peptides from samples and the isotopically labeled spiked-in peptides (Supporting Information, Table S1 & Figures S1–S4).

First, a total of 100 mg of each snap-frozen mouse tissue was weighted into 2 mL Bead Ruptor bead beating tubes prefilled with ceramic beads (Omni International, Kennesaw, GA, USA), and a homogenization buffer including protease inhibitors provided in the kit was added in a ratio of 1:2 (w/v). The samples were homogenized at 4 °C using a bead mill homogenizer (Omni Bead Ruptor 24 Elite homogenizer with a BR Cryo cooling unit, Omni International, Kennesaw, GA, USA). Crude membrane fractions were isolated from tissue homogenates as described in the manufacturer’s protocol by centrifugation and two-phase partitioning concepts. The protein concentrations of membrane fractions were determined using the Bio-Rad protein assay based on the Bradford dye-binding method (EnVision, PerkinElmer, Inc., Waltham, MA, USA). After quantifying total protein concentrations, the isolated membrane fractions were denatured, reduced, and carboxymethylated prior to digestion with LysC (Sigma-Aldrich, St. Louis, MO, USA) and TPCK-trypsin (Promega Biotech AB, Nacka, Sweden) with labeled marker peptides to produce a sample containing both digested native and labeled peptides, as previously described.^28^

The analysis was carried out on an Agilent 1290 Infinity liquid chromatography system (LC, Agilent Technologies, Waldbronn, Germany) coupled with a triple quadrupole mass spectrometer (QQQ 6495, Agilent Technologies, Santa Clara, CA, USA) with a heated electrospray ionization source in a positive ionization mode (ESI+). The injection volume was 20 μL (equal to 10 μg of digested proteins). Peptides were separated using an Agilent AdvanceBio Peptide Map 2.1 × 250 mm, 2.7 μm column (Agilent Technologies, Santa Clara, CA, USA) and eluents of water (eluent A) and acetonitrile (eluent B), both containing 0.1% (v/v) formic acid. The LC flow rate was 0.3 mL/min with the following gradient: 0–2 min: 2% B → 7%, 2–50 min: 7% B → 30%, 50–53 min: 30% B → 45%, 53–55.5 min: 45% B → 80%, 55.5–55.6 min: 80% B → 2%, 55.6–60 min: 2% B. Data were acquired using the Agilent MassHunter Workstation software (version B.06.00) and processed with the Skyline software (version 20.1).

### 2.5 Non-specific Binding of Ferulic Acid and Its Derivatives

The unbound fractions of FA, D1, and D2 were determined in the S9 subcellular fractions of the mouse liver, kidneys, and pancreas using rapid equilibrium dialysis (RED) plates (Thermo Fisher Scientific, Inc., Waltham, MA, USA). Briefly, the studied compounds were spiked at a 50 μM concentration into 100 μL of mouse homogenate S9 subcellular fraction and transferred to the reaction chamber. Homogenate dilutions were 1:4 (w/v) for the liver and kidney, and 1:20 (w/v) for the pancreas. A total of 350 μL of TBS (pH 7.4) buffer was added to the buffer chamber of the RED plate. The dialysis plate was incubated at 37 °C with shaking for 4 h. Samples of 50 μL were collected from both the reaction and buffer chambers, and equal amounts of buffer and S9 fraction were added to the collected samples, respectively, to yield identical sample matrices. The proteins were precipitated by diluting samples with 100 μL of ice-cold acetonitrile. Samples were centrifuged at 12 000 × *g* for 10 min, and the supernatants were collected for HPLC analysis.

The unbound fraction (*f*_u_,_tissue_) of the FA and its derivatives was calculated using eq 1
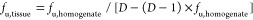
1where *D* is the dilution factor of studied tissue homogenate, and *f*_u,homogenate_ is the distribution ratio of the studied compound measured in the reaction and buffer chambers within the equilibrium dialysis assay.^29^

### 2.6 *In Vivo* Pharmacokinetics of Ferulic Acid and Its Derivatives

The pharmacokinetic study of FA, D1, and D2 was performed as previously described,^17^ in which the pharmacokinetic parameters for the plasma and brain have been reported. Briefly, the studied compounds were dissolved in a vehicle containing 0.9% (w/v) NaCl in water, and a dose of 25 μmol/kg of the FA, D1, or D2 (*n* = 3 per compound at each time point) was administered as a bolus intraperitoneal (i.p.) injection for mice. The mice were anesthetized using a mixture of ketamine (140 mg/kg) and xylazine (8 mg/kg) and sacrificed by decapitation at selected time points between 10 and 360 min. The kidney, liver, and pancreas were rapidly collected, snap-frozen on dry ice, and stored at −80 °C until analyzed.

The frozen tissues were weighed and homogenized with ultrapure water (1:3 w/v) by sonicating twice for 5 s with the SoniPrep 150 Plus disintegrator (MSE Ltd., London, UK). The proteins were precipitated by diluting homogenate 1:3 (v/v) with acetonitrile containing the internal standards—chlorzoxazone for FA and diclofenac for derivatives. Samples were then centrifuged at 14 000 *× g* for 10 min at 4 °C. Supernatants were collected and diluted further 1:1 with ultrapure water and transferred into HPLC vials for the analysis by liquid chromatography-tandem mass spectrometry (LC–MS/MS).

Drug concentrations were analyzed using the LC–MS/MS method described previously,^17^ with minor changes. Briefly, the analysis was carried out with an Agilent 1200 Series Rapid Resolution LC System with an Agilent Zorbax XBD-C18 rapid resolution high-throughput (RRHT) column (50 mm × 4.6 mm, 1.8 μm) and an Agilent 6410 triple quadrupole mass spectrometer equipped with an electrospray ionization source (Agilent Technologies, Palo Alto, CA, USA). The LC eluents were water (eluent A) and acetonitrile (eluent B), both containing 0.1% (v/v) formic acid. FA was separated with the following gradient: 0–1 min: 5% B, 1–2 min: 5% B → 90%, 2–4 min: 90% B, 4–4.1 min: 90% B → 5%, and 4.1–7 min: 5% B. For the derivatives, the gradient was as follows: 0–1 min: 10% B, 1–2 min: 10% B → 90%, 2–5 min: 90% B, 5–5.1 min: 90% B → 10%, and 5.1–8 min: 10% B. In both methods, the LC flow rate was 0.3 mL/min, the column temperature was set to 40 °C, and the sample injection volume was 5 μL.

The LC–MS/MS data acquisition for the FA was performed in a negative ion mode with the following conditions: drying gas flow 6.5 l/min with the temperature of 300 °C, nebulizer gas pressure of 20 psi, and capillary voltage of 4 000 V. MRM transitions were 193 → 149 for FA and 168 → 132 for chlorzoxazone. Fragmentor voltages were 100 and 120 V, respectively, and the collision energy was 20 V for both. For D1 and D2, the analysis was performed in a positive ion mode with the following conditions: drying gas flow of 6.5 l/min with the temperature of 300 °C, nebulizer gas pressure of 20 psi, and capillary voltage of 3 000 V. MRM transitions were 371 → 176.8 for D1, 357 → 310.9 for D2, and 296 → 250 for the diclofenac. The fragmentor voltages were 60, 40, and 100 V, and the collision energies were 16, 8, and 10 V, respectively. The data acquisition software was Agilent MassHunter Workstation software (version B.03.01), whereas Quantitative Analysis (B.09.00) software was used for data processing and analysis. The lower limit of quantification for the FA was 0.05 nmol/g and was 0.01 nmol/g for derivatives. The methods were linear, selective, accurate, and precise in the calibration range of 0.05–300 nmol/g for FA and 0.01–300 nmol/g for derivatives.

The time–concentration profiles for studied drugs were calculated using GraphPad Prism v. 5.03 software (GraphPad Software, San Diego, CA, USA). The area under curve (AUC) was calculated using the linear trapezoidal method, providing simultaneously the numeric pharmacokinetic parameters, such as *t*_max_ and *C*_max_. The half-life of elimination (t_1/2β_) was calculated from a logarithmic slope based on at least the last three detected timepoints. To compare tissue distribution of the drugs, the tissue/plasma partition coefficients (*K*_p_) were calculated for each studied tissue using eq 2
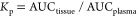
2

The unbound drug partition in studied tissues (AUC_u, tissue_) was calculated with eq 3:
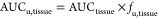
3

### 2.7 Inhibition of Lipid Peroxidation by Ferulic Acid and Derivative 1

According to the results from previous *in vitro*(18) efficacy and safety studies and *in vivo* pharmacokinetics, the efficacy of the FA and the more LAT1-specific derivative, D1 against oxidative stress and lipid peroxidation was evaluated by determining malondialdehyde (MDA) formation in mouse tissues. To induce oxidative stress, mice were administered 250 μg/kg of lipopolysaccharide (LPS) i.p. once per day for 3 consecutive days. On the 4th day, mice were anesthetized using a mixture of ketamine (140 mg/kg) and xylazine (8 mg/kg) and sacrificed by decapitation. Animals were divided into one of four treatment groups (*n* = 4 per group); (1) LPS only, (2) LPS with the FA or D1 (25 μmol/kg; i.p.) for 3 days and 120 min before the sacrifice on the 4th day (preventative efficacy), (3) LPS with the FA or D1 (25 μmol/kg; i.p.) only for the last 2 days (curative efficacy), or (4) control mice treated with 0.9% NaCl solution i.p. once per day for 3 days. After decapitation, the kidney, liver, and pancreas were rapidly collected, snap-frozen on dry ice, and stored at −80 °C until analyzed.

Pieces of snap-frozen tissues were homogenized 1:10 (w/v) in 50 mM TBS (pH 7.4) containing 0.01% butylated hydroxytoluene by sonicating samples in pulses for 5 s with a SoniPrep 150 Plus disintegrator (MSE Ltd., London, UK). The homogenates were then centrifuged at 10 000 *× g* for 20 min at 4 °C, and the supernatants were collected for the assays.

MDA was quantified by the derivatization reaction with thiobarbituric acid (TBA) (Sigma, St. Louis, MO, USA), with a previously described protocol.^30^ The analysis was carried out with an Agilent 1290 Infinity II LC System (Agilent Technologies, Santa Clara, CA, USA) combined with an Agilent 1290 Infinity II Diode Array Detector. The results were calculated as μmol of formed MDA-TBA_2_ per mg of tissue.

### 2.8 Inhibition of Prostaglandin E_2_ Production by Ferulic Acid and Derivative 1

The ability of FA and D1 to inhibit prostaglandin E_2_ (PGE_2_) production in the mouse kidney, liver, and pancreas was evaluated after the exposure of mice to LPS. The tissue homogenate supernatants (*n* = 4), prepared as described above in MDA sample preparation (Chapter 2.7), were further diluted 1:5 with 80% MeOH (v/v) solution containing the deuterated internal standard, PGE_2_-*d*_4_ (Cayman Chemical Company, Ann Arbor, MI, USA). To improve the extraction of prostaglandins and precipitation of proteins, the diluted samples were incubated overnight in a freezer (−80 °C). Subsequently, samples were centrifuged at 16 000 × *g* for 20 min at 4 °C and the supernatants were collected for the LC–MS/MS analysis.

The LC–MS/MS analysis was carried out with a previously described method^30^ using an Agilent 1290 Infinity LC System (Agilent Technologies, Waldbronn, Germany) and an Agilent 6495 triple quadrupole mass spectrometer with a heated electrospray ionization source (Agilent Technologies, Palo Alto, CA, USA). Data were collected using Agilent MassHunter Workstation software (version B.06.00) and processed with the Quantitative Analysis (B.07.00) software. The results were calculated as nmol of PGE_2_ per mg of tissue.

### 2.9 Effects of Ferulic Acid and Its Derivatives on Coagulation Parameters

To assess the safety of the LAT1 derivatives in the human blood circulation system, the basic coagulation parameters, prothrombin time (PT), activated partial thromboplastin time (APTT), thrombin time (TT), and international normalized ratio (INR) were determined from human plasma in the presence of FA, D1, and D2. The parameters were studied using a coagulometer (CoagChrom-3003 Bio-Ksel, Grudziądz, Poland), with the previously described methods.^25,31^ The following reagents were used in the basic coagulation studies: Bio-Ksel System APTT reagent and calcium chloride, Bio-Ksel PT plus reagent (thromboplastin and solvent), and thrombin (3.0 UNIH/mL), (Bio-Ksel, Grudziądz, Poland). The experiments were performed using citrated human plasma. Control samples consisting of drug solvents [distilled water and methanol mixture (1:2 v/v)] were conducted. The methods were validated using normal plasma (Bio-Ksel, Grudziądz, Poland). Coefficients of variability (CV) were counted (n = 5): CV(PT) = 5.34%, CV(APTT) = 2.61%, CV(TT) = 1.82%. Following reference values for each test were applied: PT: 9.7–14.6 s; APTT: 26.7–40.0 s; and TT: 14.0–18.0 s for 3.0 UNIH/mL of thrombin.

### 2.10 Effects of Ferulic Acid and Its Derivatives on Erythrocytes

To further evaluate the safety of derivatives in human blood, the effects of FA, D1, and D2 on hemolysis were studied using a previously described protocol.^31^ Shortly, freshly collected erythrocytes were washed three times with 1 mL of 0.9% saline (Sigma-Aldrich, Germany). Subsequently, 2% RBC suspension was prepared by diluting 1 mL of erythrocytes in 50 mL of 0.9% saline. FA, D1, and D2 (1–100 μM) were incubated with the RBC suspension at 37 °C for 1 h. Additionally, negative controls containing 0.9% NaCl and positive controls with Triton X-100 (Polish Chemical Reagents, Poland) were studied. After incubation, the samples were centrifuged at 3 000 rpm for 10 min, and the absorbance values were measured from the collected supernatants at the wavelength of 550 nm. Samples with Triton X (positive control) represented 100% hemolysis. Results of the studies are presented as a percentage of released hemoglobin. The counted CV was 8.1% (*n* = 5). Additionally, the influence of FA and its derivatives (25–100 μM) on RBC morphology was studied using a phase contrast Opta-Tech inverted microscope. The morphology was analyzed using a dedicated OptaView 7 software (Opta-Tech, Warsaw, Poland).

### 2.11 Effects of Ferulic Acid and Its Derivatives on Endothelial Cell Viability

As a third *in vitro* toxicity study in the human blood circulation system, the effects of FA, D1, and D2 on the viability of human umbilical vein endothelial cells (HUVECs; RRID CVCL_2959; Lonza, Italy; Cat. no. CC-2517) were determined using the WST-1 assay (Takara, Takara Bio Europe, Europe, Saint-Germain-en-Laye, France), according to the previously published protocol.^32^ Briefly, the cells were cultured according to the manufacturer’s guidelines with the following ingredients: EGM-2-medium + bullet kit (Lonza, Clonetics, Italy), accutase (Sigma-Aldrich, Germany), and HEPES-buffered saline solution (Lonza, Basel, Switzerland). For the assay, the cells were seeded at the density of 7 500 HUVECs per well on 96-well plates and cultured for 24 h to obtain 70% confluency. Subsequently, the cells were treated with studied compounds in multiplicate (*n* = 6–8) at the concentration of 0.1–100 μM in the growth medium for 24 h (37 °C, 5% CO_2_). Cells with untreated growth medium represented 100% viability, whereas cells treated with medium containing 0.5% (v/v) methanol served as a negative control for drug solvent. After treating cells for 24 h, medium was removed, the cells were washed with the culture medium (100 μl/well), and 100 μL of WST-1 reagent dissolved in culture medium was added into each well. The plates were incubated at 37 °C with 5% CO_2_ for 2 h, after which absorbance values were measured at the 450 nm wavelength using a microplate reader (iMARK, Bio-Rad). Additionally, the effects of treatments on HUVEC morphology were examined microscopically using a phase-contrast Opta-Tech inverted microscope equipped with OptaView 7 software (Opta-Tech, Warsaw, Poland) for image analysis.

### 2.12 Statistical Analyses

All statistical analyses were performed using the GraphPad Prism v. 5.03 software (GraphPad Software, San Diego, CA, USA). Statistical differences between groups were tested using two-way ANOVA and subsequent *post hoc* tests, whereas the variables with non-normal distributions were tested using one-way ANOVA on ranks (Kruskal–Wallis *H* test). The normal distribution of coagulation variables was verified with the Shapiro–Wilk test. The results are presented as mean ± SD, with statistically significant differences denoted by asterisks (*P < 0.05, **P < 0.01, ***P < 0.001).

## 3 Results

### 3.1 LAT1 Expression in the Mouse Brain, Kidney, Liver, and Pancreas Tissues

**Figure 2:**
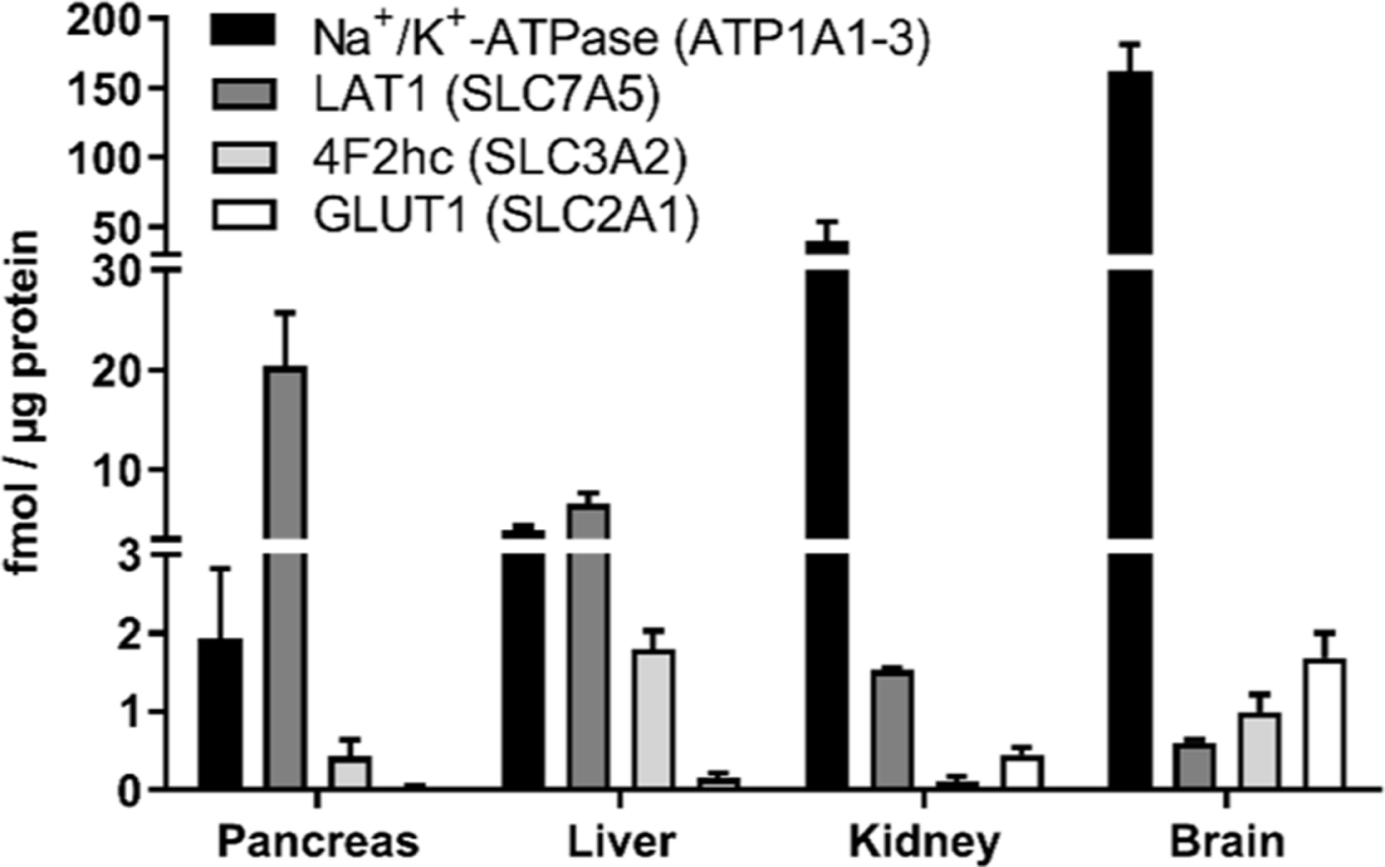
Protein expression levels of light and heavy subunits of the LAT1 (LAT1 and 4F2hc, respectively), GLUT1, and Na^+^/K^+^-ATPase alpha subunits 1–3 (ATP1A1-3) in mouse pancreas, liver, kidney, and brain tissues. The results are presented as mean ± SD (*n* = 3).

We quantified the amount of light (LAT1) and heavy (4F2hc) subunits of the LAT1 dimer in tissue samples of the mouse brain, kidney, liver, and pancreas using the LC–MS/MS technique to identify plausible tissues for increased drug accumulation via LAT1 delivery. To enhance protein level comparison between tissues, GLUT1 was also quantified simultaneously from the same samples. All transporter protein levels differed between studied tissues (Figure 2). The amount of the LAT1 light subunit was the highest in the pancreas (20.34 ± 5.35 fmol/μg protein), followed by liver (6.53 ± 1.13 fmol/μg protein) and kidney (1.53 ± 0.03 fmol/μg protein) tissues. Interestingly, the brain had the lowest protein levels of the light subunit (0.60 ± 0.04 fmol/μg protein). The heavy subunit of LAT1 was most abundant in the liver (1.79 ± 0.23 fmol/μg protein) and brain (1.00 ± 0.22 fmol/μg protein), whereas the pancreas and kidneys had smaller expressions (0.43 ± 0.21 and 0.12 ± 0.06 fmol/μg protein, respectively). The brain had the highest GLUT1 expression levels (1.69 ± 0.31 fmol/μg protein), while the kidney, liver, and pancreas showed lower levels of GLUT1 that were comparable to each other, 0.45 ± 0.09, 0.15 ± 0.07, and 0.05 ± 0.01 fmol/μg protein, respectively. The Na^+^/K^+^-ATPase alpha subunits 1–3 were also most expressed in the brain (162.05 ± 19.48 fmol/μg protein), followed again by the kidney, liver, and pancreas (40.09 ± 13.68, 3.88 ± 0.40, and 1.93 ± 0.89 fmol/μg protein, respectively).

### 3.2 Non-specific Tissue Binding of Ferulic Acid and Its Derivatives

The non-specific tissue binding of FA, D1, and D2 was studied *in vitro* in mouse liver, kidney, and pancreas S9 fractions of tissue homogenates, as drug binding decreases drug permeabilization through membranes and only free drug is pharmacologically active.^33^ The binding was studied similarly to our previous study for the mouse plasma and brain.^17^ FA had the highest free fractions in the liver, kidney, and pancreas, as most of the FA (84.94–99.30%) was unbound in studied tissues (Table 1). D2 was more bound compared to D1 in all tissues, with free portions varying between 15.01–30.52% for D2 and 28.02–46.85% for D1. Therefore, both derivatives were more bound to tissues when compared to FA, which is also in line with our previously published results on the binding of derivatives to the mouse brain homogenate.^17^ However, all the tested compounds had less non-specific binding in liver, kidney, and pancreas tissue homogenates when compared to plasma binding.

**Table 1:** Unbound Fractions (*f*_u_) of FA and Its Derivatives 1 and 2 in the Mouse Plasma, Liver, Kidney, and Pancreas Determined *In Vitro* Using the Equilibrium Dialysis Method after 4 h of Incubation^a^

	ferulic acid	derivative 1	derivative 2
*f*_u, plasma_ (%)	9.94–11.51^b^	19.66–30.62^b^	8.37–9.01^b^
*f*_u, liver_ (%)	84.94 ± 14.49	42.53 ± 5.44	23.61 ± 1.09
*f*_u, kidney_ (%)	99.13 ± 10.54	46.85 ± 5.93	30.52 ± 0.63
*f*_u, pancreas_ (%)	99.30 ± 1.96	28.02 ± 1.47	15.01 ± 2.06

a Results for plasma have been previously reported.^17^ The results are presented as mean ± SD (*n* = 3).

b *n* = 2.

### 3.3 *In Vivo* Pharmacokinetics of Ferulic Acid and Its LAT1-Utilizing Derivatives

The amounts of FA and its derivatives were quantified from mouse pancreas, kidney, and liver tissues and compared to previously published pharmacokinetic results in plasma.^17^ According to the calculated pharmacokinetic profiles and parameters (Table 2), both LAT1-utilizing derivatives were accumulating in studied tissues in higher amounts compared to the original FA. However, besides plasma, no released FA was detected in samples of animals exposed to the LAT1 derivatives. The accumulation of D1 and D2 in the studied tissues was the highest in the pancreas, where the tissue/plasma ratios (*K*_p_ values) were 4.97 and 17.65, respectively. D1 was the second most abundant in the liver, followed by kidneys, whereas D2 accumulated more in kidneys than in the liver. The tissue accumulation of FA was the highest in the liver, and as opposed to the LAT1-utilizing derivatives, the pancreas showed only small amounts of FA. These data suggest that both derivatives have significantly increased tissue delivery compared to the FA. D1 distribution follows the LAT1 expression results. D2 is transported into tissues more efficiently overall, but the delivery does not follow the LAT1 expression when comparing tissue accumulations.

**Table 2:** Pharmacokinetic Parameters for the FA and Derivatives 1 and 2 after a Bolus Injection (25 μmol/kg, i.p.) in Mice (*n* = 3)^a^

pharmacokinetic parameter	ferulic acid	derivative 1	derivative 2
AUC_total plasma_(nmol/mL × min)	591	1042 (released 507)	463 (released 75)
AUC_u, plasma_(nmol/mL × min)	63	262 (released 54)	40 (released 8)
C_max plasma_(nmol/mL)	33.2	41.9 (released 2.7)	21.1 (released 1.8)
C_max, u plasma_(nmol/mL)	3.6	10.5 (released 0.3)	1.8 (released 0.2)
t_max__plasma_ (min)	10	10 (released 30)	10 (released 10)
t_1/2β__, plasma_ (min)	N/A^b^	N/A^b^	N/A^b^
			
AUC_total pancreas_(nmol/g × min)	40.1	5 182	8 172
AUC_u, pancreas_(nmol/g × min)	40.1	1 452	1 226
*K*_p, pancreas_	0.07	4.97	17.65
AUC_u, pancreas_/AUC_u plasma_	0.64	5.54	30.65
C_max pancreas_(nmol/g)	5.4	168	253
C_max, u pancreas_(nmol/g)	5.4	47.1	38.0
t_max__pancreas_ (min)	10	10	30
t_1/2β__, pancreas_ (min)	N/A^b^	9.9	33.8
			
AUC_total liver_(nmol/g × min)	408	1 769	2 100
AUC_u, liver_(nmol/g × min)	347	752	496
K_p, liver_	0.69	1.7	4.54
AUC_u, liver_/AUC_u plasma_	5.50	2.9	12.4
C_max liver_(nmol/g)	18.0	44.3	66.4
C_max, u liver_(nmol/g)	15.3	18.8	15.7
t_max__liver_ (min)	10	10	10
t_1/2β__, liver_ (min)	11.2	11.7	13.1
			
AUC_total kidney_(nmol/g × min)	140	415	3 368
AUC_u, kidney_(nmol/g × min)	139	194	1 028
K_p, kidney_	0.24	0.40	7.27
AUC_u, kidney_/AUC_u plasma_	2.21	0.74	25.7
C_max kidney_(nmol/g)	14.4	20.4	196
C_max, u kidney_(nmol/g)	14.3	9.6	59.8
t_max__kidney_ (min)	10	10	10
t_1/2β, kidney_ (min)	3.4	11.8	8.0

a The table also includes previously reported plasma results.^17^

b N/A = not applicable (not enough data points).

**Figure 3:**
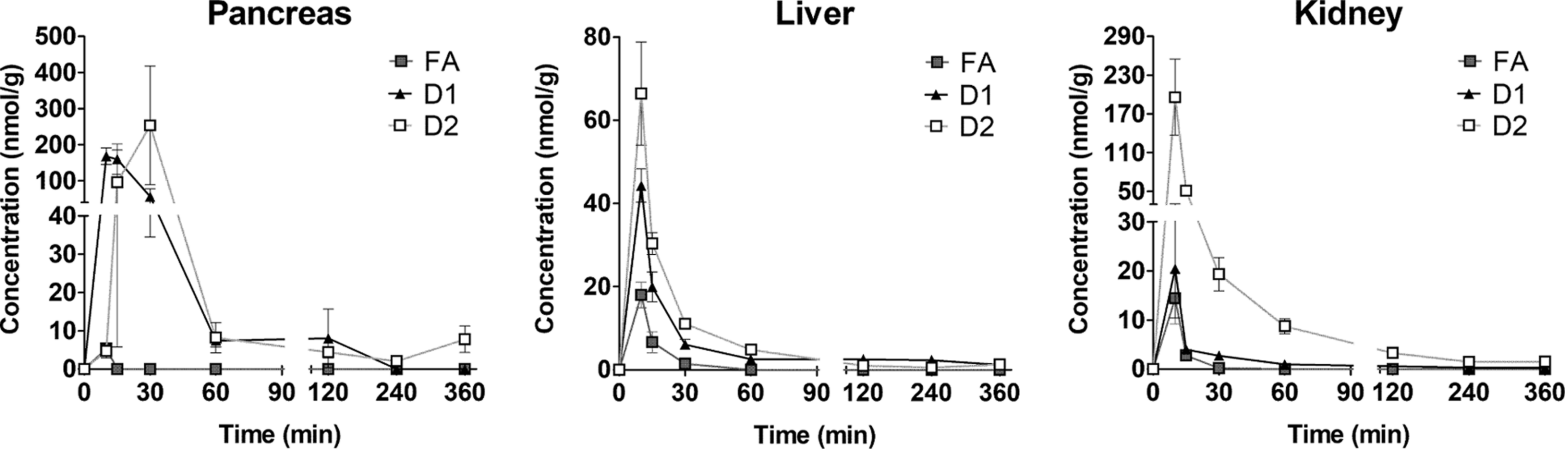
Concentration–time curves between 0 and 360 min of FA, LAT1-utilizing derivative 1 (D1), and derivative 2 (D2) in mouse pancreas, liver, and kidney tissues (*n* = 3 for each timepoint) after a single dose (25 μmol/kg, i.p.). Concentrations per each timepoint are represented as mean ± SD.

The peak concentration (*C*_max_) was the highest in the pancreas with both D1 and D2 (168 and 253 nmol/g, respectively). With D1, also the unbound peak concentration (*C*_max, u_) was the highest in the pancreas. However, due to the higher unspecific binding of D2 in the pancreas, *C*_max, u_ was the highest in kidneys. The accumulation of derivatives and FA was rapid in all studied tissues (Figure 3), as the peak concentrations were observed at the 10 min timepoint, excluding the D2 in the pancreas, which had the *t*_max_ at 30 min (Table 2). Also, the elimination half-lives of FA and its derivatives were similar between tissues, and differences were only seen with the kidney and pancreas. In the pancreas, the half-life followed the trend of slower *t*_max_ as D2 had a half-life of 33.8 min (Table 2, Figure 3), whereas D1 was eliminated more rapidly (*t*_1/2β_ = 9.9 min). In the kidney, the FA had the fastest elimination with the half-life of 3.4 min, whereas D1 and D2 remained there longer (*t*_1/2β_ = 11.8 min and 8 min, respectively).

### 3.4 Ability of FA and Derivative 1 to Inhibit Lipid Peroxidation and Prostaglandin Synthesis

**Figure 4:**
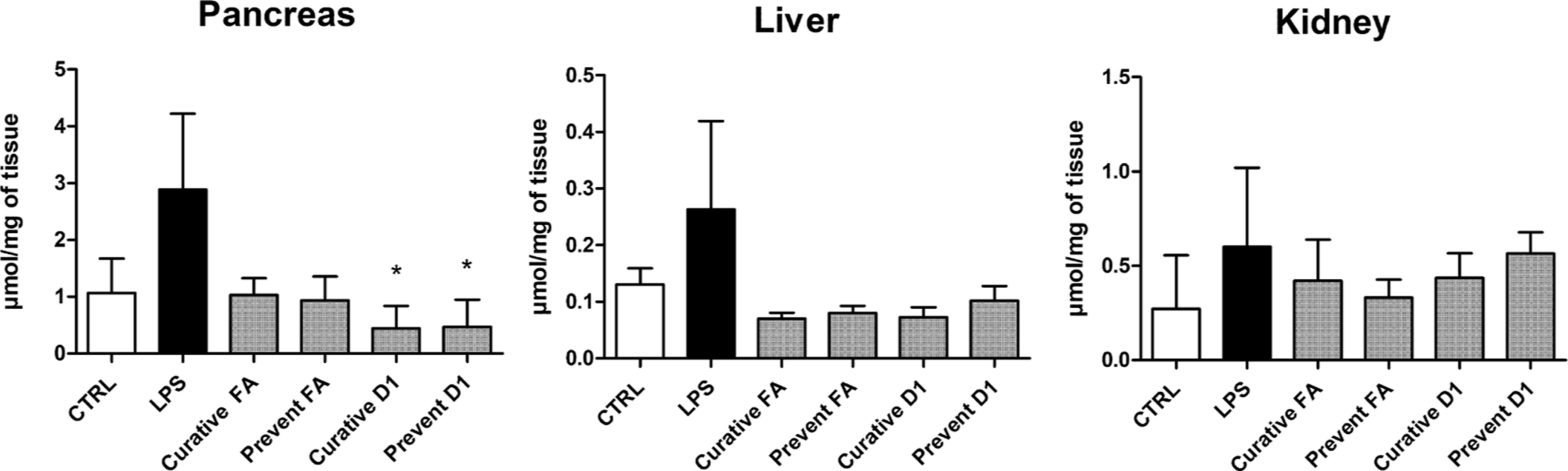
Formation of MDA in mouse pancreas, liver, and kidney tissues after LPS induction (250 μg/kg, i.p) and treatment with FA and its derivative 1 (D1) (25 μmol/kg; i.p). Control (CTRL) mice were treated with NaCl solution without LPS induction. The results are presented as mean ± SD (*n* = 3–4). An asterisk denotes statistical significance in comparison to LPS-induced (*p < 0.05) using one-way ANOVA on ranks (Kruskal–Wallis H test).

As D1 presented more LAT1-selective drug delivery, it was selected for further *in vivo* efficacy studies. The antioxidative efficacy of the FA and D1 was studied from the peripheric LPS-induced mouse tissues by measuring MDA, the final product of lipid peroxidation. The LPS induction elevated MDA formation in the pancreas (2.89 ± 1.33 with LPS vs 1.07 ± 0.60 μmol/mg in control) (Figure 4). There, both FA and D1 were able to reduce the LPS-induced formation (Figure 4). The FA treatment decreased MDA formation with both curative and preventative administrations (1.04 ± 0.29 and 0.94 ± 0.41 μmol/mg, respectively). The antioxidative effect was even more pronounced with D1 since MDA levels were significantly lower upon both curative and preventative treatments (0.44 ± 0.39 and 0.46 ± 0.47 μmol/mg, respectively). In liver and kidney tissues, the MDA levels were many times lower compared to those in the pancreas, ranging between 0.06–0.44 and 0.07–1.06 μmol/mg, respectively. In the liver, the MDA levels corresponded to the pancreatic results (Figure 4), but due to high variation in the LPS-induced group, there were no significant differences. Subsequently, the increase of MDA formation in the kidney by the LPS induction was insignificant (0.60 ± 0.42 with LPS vs 0.27 ± 0.28 μmol/mg in control). There, the FA and D1-treated groups had MDA levels between the control and LPS-induced, with no significant differences.

**Figure 5:**
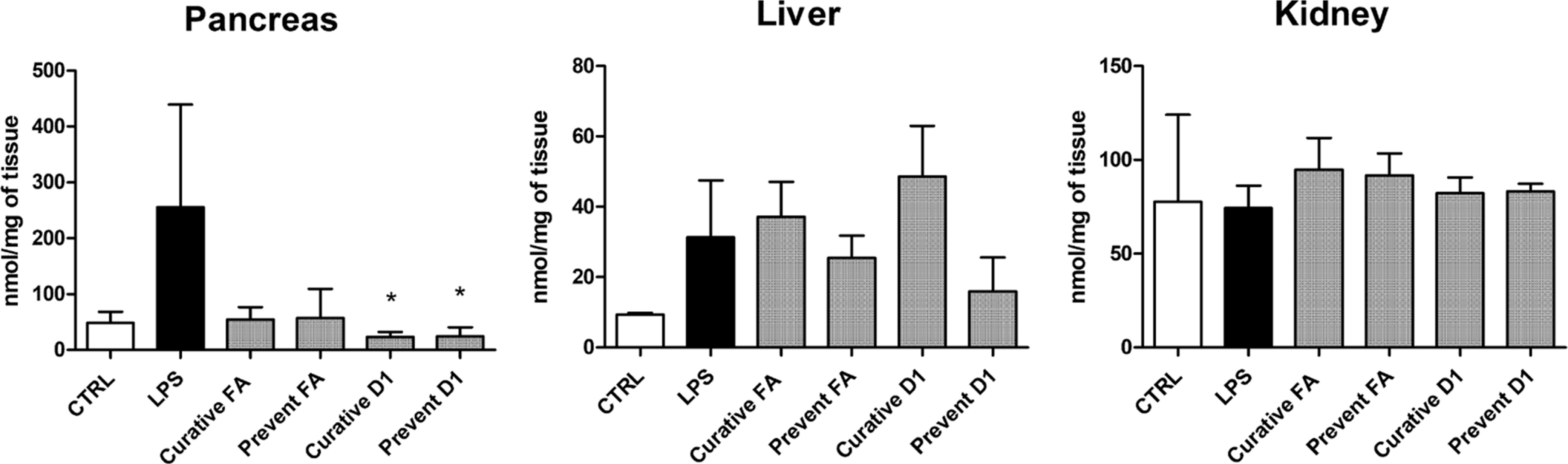
Production of PGE_2_ in the mouse pancreas, liver, and kidney after LPS induction (250 μg/kg, i.p) and treatment with FA and its derivative 1 (D1) (25 μmol/kg; i.p). CTRL mice were treated with NaCl solution without LPS induction. The results are presented as mean ± SD (*n* = 3–4). An asterisk denotes statistical significance in comparison to LPS-induced (*p < 0.05) using one-way ANOVA on ranks (Kruskal–Wallis H test).

In addition to the MDA, PGE_2_ was also quantitated from the same pancreas, liver, and kidney homogenates. PGE_2_ production was increased by LPS in the pancreas (255.60 ± 183.79 nmol/mg with LPS vs 48.56 ± 19.86 nmol/mg in control) (Figure 5). The mice treated with D1 had significantly lower PGE_2_ concentrations in the pancreas when compared to those exposed only to LPS with both curative and preventative treatments (23.51 ± 8.81 and 24.51 ± 15.99 nmol/mg, respectively) (Figure 5). The FA treatment also lowered PGE_2_, but to a lesser extent (54.68 ± 22.02 nmol/mg with curative, 57.56 ± 51.63 nmol/mg with preventative treatment). In the kidney, the PGE_2_ levels were almost identical with all studied groups, with PGE_2_ concentrations varying from 39.25 to 129.20 nmol/mg (Figure 5). In the liver, PGE_2_ production was the lowest of all studied tissues even after the LPS induction (31.27 ± 16.20 nmol/mg with LPS vs 9.34 ± 0.49 nmol/mg in control). The preventative treatments with both FA and D1 were able to lower PGE_2_ production slightly (25.41 ± 6.35 and 15.95 ± 9.61 nmol/mg, respectively), whereas the curative treatments had higher PGE_2_ levels (37.07 ± 10.02 and 48.55 ± 14.50 nmol/mg, respectively).

In conclusion, D1 had favorable effects on both MDA and PGE_2_ formation with both curative and preventative treatments in the pancreas. Similar effects were also seen with FA, to a lesser extent. In the liver and kidney, the LPS-model did not distinguish the studied compounds by their efficacy.

### 3.5 Effects of Ferulic Acid and Its Derivatives on Human Plasma Hemostasis

**Figure 6:**
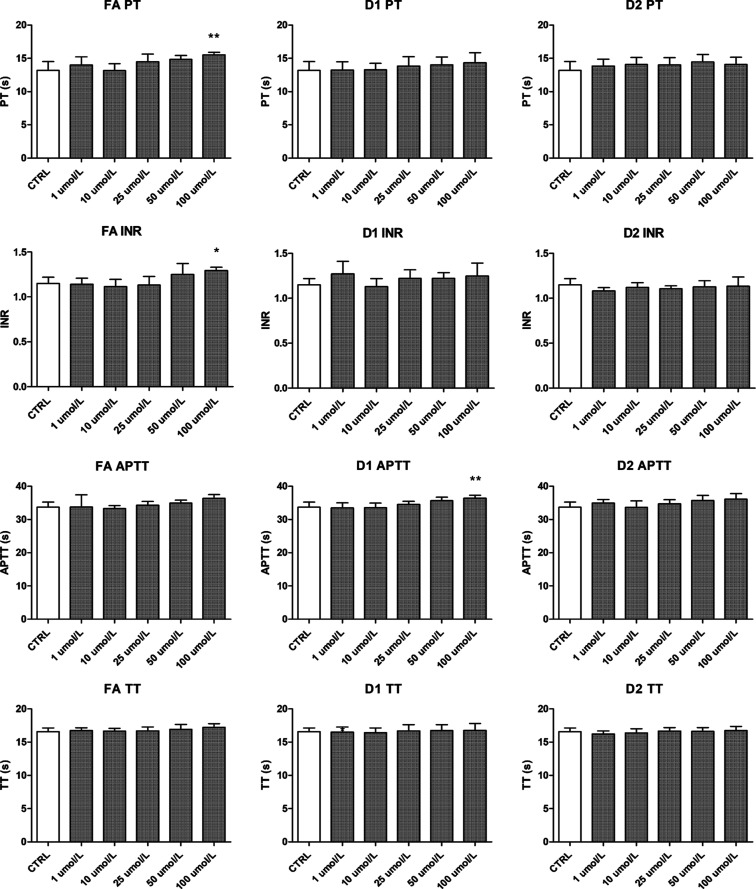
Effects of FA and its derivatives (D1 and D2) on PT, INR, APTT, and TT in human plasma. The data are expressed as mean ± SD, *n* = 5. Asterisk denotes statistical significance in comparison to the CTRL (*p < 0.05; **p < 0.01) with two-way ANOVA (homogeneity of variance; two independent variables: concentration and compound).

To evaluate the derivatives’ safety in the human blood circulation system, the effects of FA, D1, and D2 on the coagulation parameters were evaluated as part of the hemocompatibility studies. The original drug FA increased the PT significantly (15.5 ± 0.4 s vs 13.2 ± 1.3 s for control) at the highest 100 μM concentration (Figure 6). Subsequently, FA also increased INR, whereas neither derivative significantly affected PT or INR at the studied concentration range. However, D1 prolonged the APTT at the 100 μM concentration, whereas lower concentrations did not have significant effects. None of the tested compounds affected the TT. Collectively, all studied compounds were found hemocompatible at the 1–50 μM concentration range.

### 3.6 Effects of Ferulic Acid and Its Derivatives on the Integrity of RBCs

**Figure 7:**
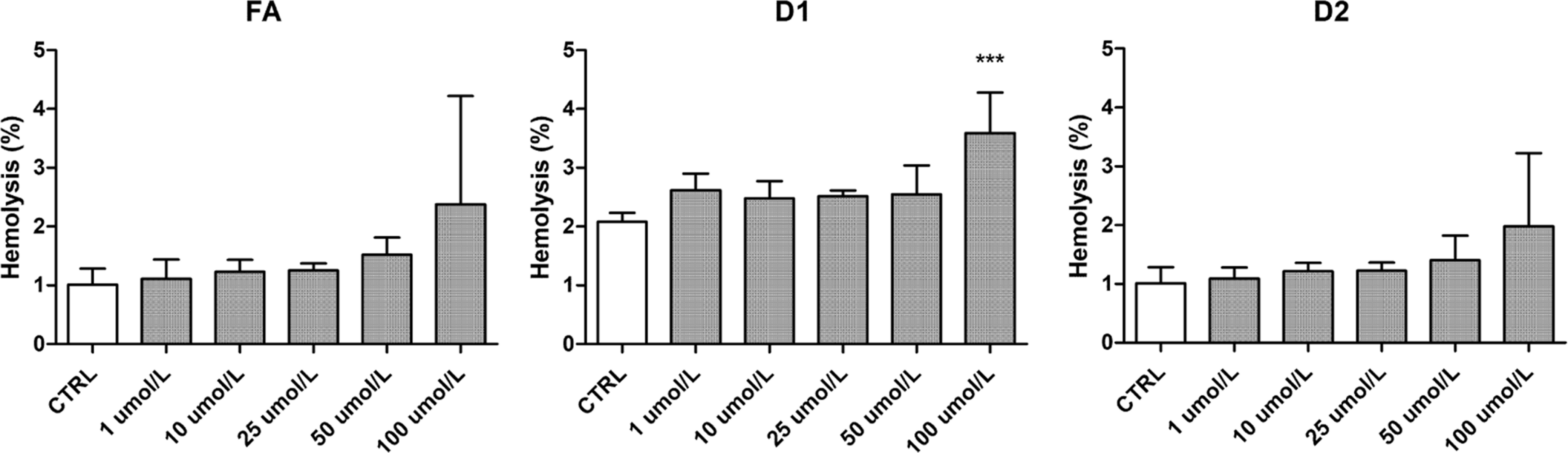
Effects of FA and derivatives 1 and 2 (D1 and D2) on the hemolysis rate after 1 h of incubation with human RBCs with concentrations 1–100 μmol/L. The results are expressed as mean ± SD (*n* = 4). *** denotes statistical significance (*p* < 0.001) in comparison to the CTRL using one-way ANOVA on ranks (Kruskal–Wallis H test).

The effects of FA, D1, and D2 on the integrity of the human RBC membrane and subsequent hemolysis were studied with an RBC lysis assay to further assess their hemocompatibility. The increasing concentration of any studied compound increased the hemolysis slightly (Figure 7). Statistically, only D1 exerted a significant effect on the hemolysis rate at the highest tested concentration (100 μM), with the hemolysis rate increasing from 2.08 ± 0.15% (control) to 3.58 ± 0.69%. The FA and D2 also had increased hemolysis with a 100 μM concentration when compared to the corresponding controls (2.38 ± 1.84% and 1.97 ± 1.25%, respectively), but due to high variance, it did not reach statistical significance.

**Figure 8:**
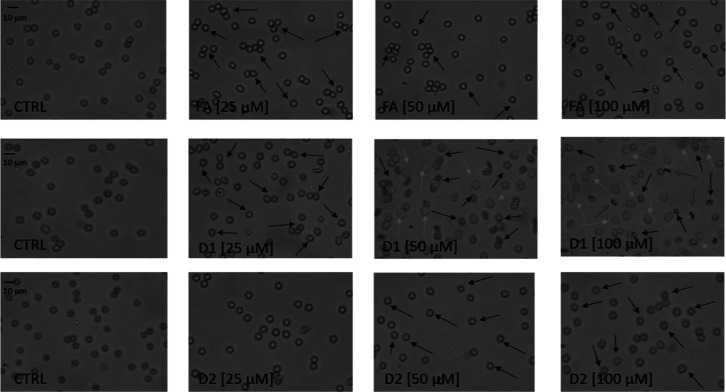
Effects of FA and its derivatives (D1 and D2) at the concentrations 25–100 μM on human erythrocyte morphology. Representative phase-contrast images are shown with 400-fold magnification. CTRL–control samples; morphologically changed erythrocytes are marked with arrows: echinocytes—black arrows, macrocytes—green arrows, while stomatocytes are marked with red arrows.

The erythrocyte morphology after exposure to FA and its derivatives was also microscopically evaluated. The analysis showed that FA and D1 contributed to the extensive formation of echinocytes at a 25–100 μM concentration (Figure 8). Similar morphological changes were also observed with the D2 at 50–100 μM (Figure 8). In addition to the echinocyte formation, D1 induced an extensive anisocytosis manifested by the formation of macrocytes at 50–100 μM (Figure 8). Furthermore, few stomatocytes were detected with D1 at the highest 100 μM concentration (Figure 8).

### 3.7 Viability Effects of Ferulic Acid and Its Derivatives on Human Endothelial Cells

**Figure 9:**
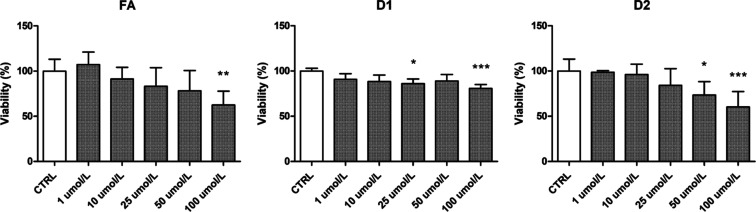
Effects of FA and its derivatives (D1 and D2) on HUVEC viability. The results of viability assays are expressed as a percentage of the control samples that represented 100% viability. The results are presented as mean ± SD (*n* = 6–8). Asterisks denote statistical significance (*p < 0.05; **p < 0.01; ***p < 0.001) in comparison to the CTRL using one-way ANOVA on ranks (Kruskal–Wallis H test).

The effects of FA, D1, and D2 on the viability of HUVECs were evaluated using the WST-1 assay, which follows the mitochondrial activity of living cells. While gradually decreasing the viability of the cells, the FA had no statistically significant effect on the HUVEC viability at 1–50 μM concentrations, but at 100 μM, it significantly reduced the cell viability down to 62.62 ± 15.02% (Figure 9). D2 had the most profound effect on HUVEC viability since it reduced the cell viability down to 73.45 ± 14.69% at 50 μM concentration and further to 60.27 ± 16.95% at 100 μM concentration. D1 exerted clearly more favorable effects on HUVEC viability when compared to FA and D2 since it diminished the viability at 50 and 100 μM concentrations only to 88.98 ± 7.27% and 80.67 ± 4.26%, respectively.

**Figure 10:**
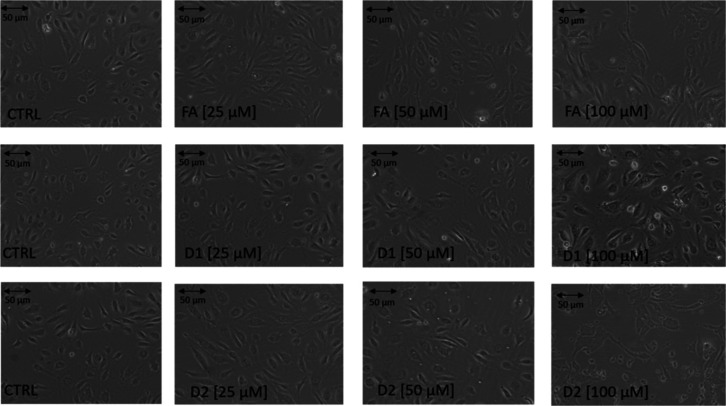
Effects of FA and its derivatives 1 and 2 (D1 and D2) on HUVEC morphology. HUVECs were cultured in the presence of tested compounds at concentrations of 1–100 μM; cultures in medium alone were also used as CTRL. Representative phase-contrast cell images of control samples and cells treated with 25, 50, and 100 μM of compounds are shown after 24 h of incubation (100-fold magnification).

The effects of FA and its derivatives on HUVEC morphology were also microscopically evaluated (Figure 10). Exposure of HUVECs to 25–50 μM of FA did not change cell morphology, whereas, at the 100 μM concentration, more elongated cells were seen (Figure 10). D1 did not cause significant changes in HUVEC morphology either at 25–50 μM concentrations (Figure 10), although slightly more rounded, bright dead cells were observed at the highest 100 μM concentration (Figure 10). D2 reduced the number of viable cells and cell density as well as caused an irregular shape with cytoplasm leakage at the 100 μM concentration, with the first brighter cells appearing already at a 50 μM concentration (Figure 10). Together with the data from the WST-1 assay, our results suggest that D2 reduces endothelial cell viability starting from a 50 μM concentration, whereas D1 had the most favorable effects on cell viability at the whole studied concentration range.

## 4 Discussion

In the present study, we observed substantially high protein expression of the LAT1 light subunit in the mouse pancreas, which supports our previous finding about the high pancreatic accumulation of LAT1-utilizing prodrugs of perforin inhibitors.^24^ However, the heavy subunit 4F2hc had lower pancreatic expression levels similar to the other studied tissues, namely the liver, kidney, and brain. According to the Human Protein Atlas (www.proteinatlas.org), 4F2hc is highly expressed in endocrine cells of the human pancreas but not found in exocrine cells, whereas LAT1 is abundant in both cell types with medium expression.^34^ The present study cannot distinguish cell types in tissues but gives average expression in whole tissue. As the functional transporter requires both subunits, the heavy subunit appears to be the limiting factor for the active protein in the mouse pancreas, as well as in the kidney and liver. However, similar variance in the light and heavy subunit ratio has also been observed previously, with both cell samples and brain slices.^35,36^ 4F2hc may suffer from feeble extraction and digestion power when compared to the LAT1. However, due to the limited variance in the protein expression result for each replicate, the reason of varying ratios is yet unknown. However, differences in the protein expression per total protein of sample may also result from the heterogenicity of tissue samples, as the protein expression in the present study was analyzed from whole-tissue homogenates. It is known that the expression of LAT1 subunits varies between cell types in many heterogeneous tissues, for example, in the brain.^34^ Consequently, in our whole-tissue comparison, the brain had the lowest LAT1 expression, although it is a known LAT1 target tissue due to its ability to transport drugs across the BBB.^37^ To support the comparison of protein expression levels between tissues, we included the GLUT1 and a common Na^+^/K^+^-ATPase alpha subunit (1–3) peptide in the analysis, as they are conserved transmembrane proteins expressed in almost all tissues to some extent.^38^ In our study, the GLUT1 expression was the highest in the brain, followed by the kidney, liver, and pancreas. The results are in line with the RNA levels reported in the Human Protein Atlas, whereas the protein expression in kidneys has been reported to be exceeding the expression in the brain.^34^ This is again likely due to the comparison of expression levels per total protein amounts of tissues, as according to Human Protein Atlas, the GLUT1 in the kidney is localized at the collecting ducts and distal tubules. The subunits of the Na^+^/K^+^-ATPases are known to be highly expressed in the human brain and kidney, which also corroborates our results from the mouse. As the GLUT1 and Na^+^/K^+^-ATPase results follow previous findings, the comparison of LAT1 protein expression between tissues can be approximated. However, the interspecies differences are also possible when comparing human and mouse protein expressions, and therefore, the extent of the LAT1 expression should also be validated in human context.

As a validation for the LAT1 expression results by the activity, the *in vivo* pharmacokinetic results of the present study were in line with the widespread expression of the LAT1 in mouse tissues. The pancreas showed the highest accumulation of LAT1-utilizing derivatives D1 and D2, with AUCs increasing 129.2- and 203.8-times higher in comparison to the original FA, respectively. We have previously reported similar pancreatic accumulation with the LAT1-utilizing prodrugs of perforin inhibitors, which showed increased delivery into the pancreas along the brain.^24,30^ In addition to our prodrugs, a LAT1-targeting positron emission tomography-probe, 5-(2-^18^F-fluoroethoxy)-l-tryptophan, has been reported to accumulate significantly in wild-type mouse pancreas.^39^ Furthermore, the LAT1-utilizing drug levodopa has been observed to accumulate into both mouse and human pancreas, supporting the common LAT1-mediated delivery between species.^40^ These findings with multiple LAT1-targeted compounds combined with pharmacoproteomics suggest that the LAT1-mediated drug targeting also increases pancreatic drug delivery greatly. Consequently, as the LAT1 was expressed in all the studied tissues, the drug distribution into all these tissues was higher when compared to the original FA. Increases in the liver and kidney accumulation were in line with the results of the protein expression. However, D2 accumulated noticeably more in the kidney when compared to D1. One possible reason for high kidney accumulation is another transportation mechanism, which is more prominent for D2. The cellular uptake mechanisms have previously been studied with these two derivatives in vitro.^18^ There, we observed that D2 also utilized another non-validated transport mechanism, especially with high concentrations, although the LAT1 was the main transport mechanism for both derivatives with higher affinity. According to our previous studies, the secondary transport mechanism of D2 could be one of organic anion transporting polypeptides or organic anion transporters since the uptake of similar prodrugs has been observed to be sensitive for non-selective inhibition by probenecid.^17,41^ The previous uptake mechanism studies with the FA derivatives have shown that the affinity of D2 toward LAT1 is over 20-times higher when compared to the secondary mechanism, while the overall uptake speed of D2 is 1.5-times faster when compared to D1.^18,42^ However, in the present study, with a single high-dose administration, it is possible that other low-affinity transporters along LAT1 are also affecting the distribution of the compounds.

Besides kidney accumulation, the two FA derivatives had very similar pharmacokinetic properties. Main differences were noticed in the plasma, where D1 was more abundant, and in the pancreas, where the accumulation and elimination of D2 were slower than those of D1. Overall, the AUC trends were similar in both derivatives, but the higher AUC of D1 in the plasma decreases the tissue/plasma distribution coefficients (*K*_p_), separating the two derivatives. According to the *K*_p_ values, D2 accumulates particularly into the tissues, while also D1 is more present in studied tissues when compared to the original FA. As a highlight, the accumulations of both D1 and D2 were greatly increased in the pancreas (*K*_p_ values of 4.97 and 17.65, respectively) when compared to FA (*K*_p_ = 0.07). Despite the increased delivery into tissues, no released FA was detected during the pharmacokinetic study. This is in line with our previous *in vitro* bioconversion studies, where neither D1 nor D2 released FA in the mouse liver.^17^ Therefore, it is possible that the LAT1-utilizing derivatives of FA are eliminated by a phase II conjugation, similar to the sugar esters of FA studied previously.^43^

In the present study, the efficacy of FA and the more LAT1-selective derivative D1 against lipid peroxidation and prostaglandin synthesis in peripheric tissues was studied *in vivo* by quantitation of MDA and PGE_2_ in the mouse pancreas, liver, and kidney. The LAT1-utilizing derivatives of FA have been previously reported to show antioxidative efficacy *in vitro*, although no released FA has been detected.^18^ This suggests that derivatives are active as such and are not regarded as prodrugs. Comparably, we did not detect any released FA in mouse tissues in the pharmacokinetic study with derivatives. However, after promoting oxidative stress with the LPS, we were able to see significantly reduced levels of MDA and PGE_2_ in the pancreas following the treatment by D1. According to MDA and PGE_2_, there were no differences in whether the treatment started on the same time as LPS induction (preventative efficacy) or afterward (curative efficacy). This is beneficial, as the treatments can also revert the changes caused by oxidative stress afterward, not only preventatively. Unfortunately, LPS induction did not result in significant changes with MDA and PGE_2_ in the liver and kidneys. This might be due to a relatively low LPS dosage (0.25 mg/kg), as many times higher doses (10–15 mg/kg) have been used in the induction of acute inflammation in the kidney and liver with mice.^44−46^ However, higher LPS doses also result in the occasional death of mice in studies spanning longer,^47^ which is not favorable when the drug efficacy is studied against low-level inflammation and oxidative stress. In addition to the LPS dose, the prolonged time (24 h) between the last LPS dose and sacrifice may diminish the MDA and PGE_2_ levels. In the previous studies on time-dependent expression of PGE_2_ with mice, the PGE_2_ plasma concentration returned to baseline in 6–12 h after the LPS administration.^44^ This supports our present findings about low levels of MDA and PGE_2_ in highly perfused tissues such as the liver and kidney. Altogether, to evaluate the efficacy differences between LAT1 derivatives and FA, further studies on action mechanisms against oxidative stress and inflammation should be carried out with more accurate disease models. Furthermore, as the LAT1 is also known to be highly upregulated in human pancreatic ductal adenocarcinoma along with other cancer types,^48^ the pancreatic LAT1 delivery could also be aimed against pancreatic cancer. This, however, requires anti-cancer properties from the drug as well, which may be insufficient with the LAT1 derivatives of FA.

The hemocompatibility of FA and its derivatives in human plasma and erythrocytes was studied to estimate the safety of these LAT1-utilizing derivatives as potential clinical treatments for the first time in humans. According to the results, the erythrocytes tolerated the studied compounds well at 1–50 μM concentrations. At the same concentration range, no significant changes in coagulation parameters were observed either. At the 100 μM concentration, only D1 increased the hemolysis rate significantly. However, since the *in vitro* hemolysis rate was always below 10%, our results suggest that the FA and its derivatives can be regarded as non-hemolytic.^49^ In addition to the slightly increased hemolysis, formation of echinocytes, macrocytes, and stomatocytes were observed with D1 beginning at a 25 μM concentration. With the FA and D2, only minor morphological changes were detected at 25–100 and 50–100 μM, respectively. However, the unpredictable transformation of erythrocytes to echinocytes and stomatocytes also occurs in the healthy blood stream, resulting from various factors, including changes in ion strength, pH, or ATP depletion.^50,51^ Therefore, these changes may not cause clinically significant harm. Additionally, the effects on coagulation parameters and hemolysis at the high concentrations can likely be regarded as irrelevant for safety as the peak concentrations in plasma after a high bolus dose were still lower for both the FA and D1 (33.2 and 41.9 μM, respectively), and the tissue distribution and elimination were rapid.

Lastly, the effects of compounds on the viability of HUVECs were studied to further estimate the safety of the LAT1-utilizing derivatives of FA in the human blood circulation system. In contrast to the erythrocyte and coagulation effects, D1 only had a modest effect on HUVEC viability, whereas D2 decreased cell viability the most. These effects are good to recognize, as they show that similar derivatives may have opposite effects on different tissues, cell types, and plasma parameters. However, the significant changes in cell viability were once again observed only at higher concentrations (≥50 μM) and are not likely to arise with therapeutic doses, as much lower concentrations were seen as peak concentrations in the pharmacokinetic study. Therefore, taking the data together, the LAT1-utilizing derivatives of FA can be considered safe in the human blood circulation system with concentrations below 50 μM, but more comprehensive toxicity studies (e.g. *in vivo*) should be carried out in the future.

## 5 Conclusions

In conclusion, the present study corroborates that LAT1 is present in the mouse pancreas. LAT1 enhances the distribution of LAT1-utilizing derivatives of FA, as the delivery of both derivatives increases in all tissues, especially in the pancreas. With its efficient delivery into the pancreas, the LAT1-utilizing D1 was also able to reduce lipid peroxidation and prostaglandin synthesis induced by the LPS. This activity is likely from the derivative on its own, as no released FA was detected during the pharmacokinetic study. Like the FA, both LAT1-utilizing derivatives were hemocompatible in human plasma at concentrations ≤50 μM. As the LAT1-utilizing derivatives have also previously improved delivery into the brain and shown antioxidative efficacy with glial cells,^17,18^ they can now be considered as hemocompatible multitargeting drugs. Therefore, the LAT1 can also be regarded as a potential multitargeting drug transporter, which is beneficial in the development of therapeutic agents targeting both the pancreas and the brain. However, the magnitude of LAT1-mediated drug delivery in the human pancreas should still be verified more comprehensively in the future.

## Supporting Information Available

Peptide sequences and MRM parameters used for the *in vivo* quantitative proteomics method and example chromatograms of native and labeled peptides (PDF)

## Abbreviations

AD: Alzheimer’s disease
Aβ: beta-amyloid
ACN: acetonitrile
APTT: partially activated thromboplastin time
BBB: blood–brain barrier
DM: diabetes mellitus
FA: ferulic acid
HPLC: high-performance liquid chromatography
HUVEC: human umbilical vein endothelial cells
INR: international normalized ratio
LAT1: L-type amino acid transporter 1
LC–MS/MS: liquid chromatography–tandem mass spectrometry
LPS: lipopolysaccharide
MDA: malondialdehyde
MRM: multiple reaction monitoring
PD: Parkinson’s disease
PGE_2_: prostaglandin E_2_
PT: prothrombin time
RBCs: red blood cells
ROS: reactive oxygen species
TBA: thiobarbiturate acid
TBS: tris-buffered saline
TT: thrombin time

## References

[ref1] Stringer D. M.; Zahradka P.; Taylor C. G. Glucose Transporters: Cellular Links to Hyperglycemia in Insulin Resistance and Diabetes. Nutr. Rev. 2015, 73, 140–154. 10.1093/nutrit/nuu012. 26024537

[ref2] Rudich A.; Tirosh A.; Potashnik R.; et al. Prolonged Oxidative Stress Impairs Insulin-Induced GLUT4 Translocation in 3T3-L1 Adipocytes. Diabetes 1998, 47, 1562–1569. 10.2337/diabetes.47.10.1562. 9753293

[ref3] Luc K.; Schramm-Luc A.; Guzik T. J.; et al. Oxidative Stress and Inflammatory Markers in Prediabetes and Diabetes. J. Physiol. Pharmacol. 2019, 70, 111–113. 10.26402/jpp.2019.6.01. 32084643

[ref4] Cheong J. L. Y.; de Pablo-Fernandez E.; Foltynie T.; et al. The Association Between Type 2 Diabetes Mellitus and Parkinson’s Disease. J. Parkinson’s Dis. 2020, 10, 775–789. 10.3233/jpd-191900. 32333549 PMC7458510

[ref5] Yang Y.; Song W. Molecular links between Alzheimer’s disease and diabetes mellitus. Neuroscience 2013, 250, 140–150. 10.1016/j.neuroscience.2013.07.009. 23867771

[ref6] Saeedi P.; Petersohn I.; Salpea P.; et al. Global and regional diabetes prevalence estimates for 2019 and projections for 2030 and 2045: Results from the International Diabetes Federation Diabetes Atlas, 9th edition. Diabetes Res. Clin. Pract. 2019, 157, 107843 10.1016/j.diabres.2019.107843. 31518657

[ref7] Sgarbossa A.; Giacomazza D.; Di Carlo M. Ferulic Acid: A Hope for Alzheimer’s Disease Therapy from Plants. Nutrients 2015, 7, 5764–5782. 10.3390/nu7075246. 26184304 PMC4517023

[ref8] Kumar N.; Pruthi V. Potential Applications of Ferulic Acid from Natural Sources. Biotechnol. Rep. 2014, 4, 86–93. 10.1016/j.btre.2014.09.002. PMC5466124 28626667

[ref9] Ghosh S.; Chowdhury S.; Sarkar P.; et al. Ameliorative Role of Ferulic Acid against Diabetes Associated Oxidative Stress Induced Spleen Damage. Food Chem. Toxicol. 2018, 118, 272–286. 10.1016/j.fct.2018.05.029. 29758315

[ref10] Choi R.; Kim B. H.; Naowaboot J.; et al. Effects of Ferulic Acid on Diabetic Nephropathy in a Rat Model of Type 2 Diabetes. Exp. Mol. Med. 2011, 43, 676 10.3858/emm.2011.43.12.078. 21975281 PMC3256295

[ref11] Nagai N.; Kotani S.; Mano Y.; et al. Ferulic Acid Suppresses Amyloid β Production in the Human Lens Epithelial Cell Stimulated with Hydrogen Peroxide. BioMed Res. Int. 2017, 2017, 5343010 10.1155/2017/5343010. 28409157 PMC5376927

[ref12] Cho J. Y.; Kim H. S.; Kim D. H.; et al. Inhibitory Effects of Long-Term Administration of Ferulic Acid on Astrocyte Activation Induced by Intracerebroventricular Injection of β-Amyloid Peptide (1-42) in Mice. Prog. Neuro-Psychopharmacol. Biol. Psychiatry 2005, 29, 901–907. 10.1248/bpb.27.120. 15970368

[ref13] Cheignon C.; Tomas M.; Bonnefont-Rousselot D.; et al. Oxidative stress and the amyloid beta peptide in Alzheimer’s disease. Redox Biol. 2018, 14, 450–464. 10.1016/j.redox.2017.10.014. 29080524 PMC5680523

[ref14] Hampel H.; Hardy J.; Blennow K.; et al. The Amyloid-β Pathway in Alzheimer’s Disease. Mol. Psychiatry 2021, 26, 5481–5503. 10.1038/s41380-021-01249-0. 34456336 PMC8758495

[ref15] Li X.; Zhang J.; Rong H.; et al. Ferulic Acid Ameliorates MPP+/MPTP-Induced Oxidative Stress via ERK1/2-Dependent Nrf2 Activation: Translational Implications for Parkinson Disease Treatment. Mol. Neurobiol. 2020, 57, 2981–2995. 10.1007/s12035-020-01934-1. 32445087

[ref16] Zhao Z.; Moghadasian M. H. Chemistry, Natural Sources, Dietary Intake and Pharmacokinetic Properties of Ferulic Acid: A Review. Food Chem. 2008, 109, 691–702. 10.1016/j.foodchem.2008.02.039. 26049981

[ref17] Puris E.; Gynther M.; Huttunen J.; et al. L-Type Amino Acid Transporter 1 Utilizing Prodrugs of Ferulic Acid Revealed Structural Features Supporting the Design of Prodrugs for Brain Delivery. Eur. J. Pharm. Sci. 2019, 129, 99–109. 10.1016/j.ejps.2019.01.002. 30625368

[ref18] Montaser A.; Huttunen J.; Ibrahim S. A.; et al. Astrocyte-Targeted Transporter-Utilizing Derivatives of Ferulic Acid Can Have Multifunctional Effects Ameliorating Inflammation and Oxidative Stress in the Brain. Oxid. Med. Cell. Longevity 2019, 2019, 3528148 10.1155/2019/3528148. PMC6877910 31814871

[ref19] Kanai Y.; Segawa H.; Miyamoto K. I.; et al. Expression Cloning and Characterization of a Transporter for Large Neutral Amino Acids Activated by the Heavy Chain of 4F2 Antigen (CD98). J. Biol. Chem. 1998, 273, 23629–23632. 10.1074/jbc.273.37.23629. 9726963

[ref20] Uchino H.; Kanai Y.; Kim D. K.; et al. Transport of Amino Acid-Related Compounds Mediated by L-Type Amino Acid Transporter 1 (LAT1): Insights into the Mechanisms of Substrate Recognition. Mol. Pharmacol. 2002, 61, 729–737. 10.1124/mol.61.4.729. 11901210

[ref21] Takahashi Y.; Nishimura T.; Higuchi K.; et al. Transport of Pregabalin Via L-Type Amino Acid Transporter 1 (SLC7A5) in Human Brain Capillary Endothelial Cell Line. Pharm. Res. 2018, 35, 264 10.1007/s11095-018-2532-0. PMC6208607 30374619

[ref22] Kobayashi N.; Okazaki S.; Sampetrean O.; et al. CD44 variant inhibits insulin secretion in pancreatic β cells by attenuating LAT1-mediated amino acid uptake. Sci. Rep. 2018, 8, 2785 10.1038/s41598-018-20973-2. 29434323 PMC5809395

[ref23] Cheng Q.; Beltran V. D.; Chan S. M. H.; et al. System-L amino acid transporters play a key role in pancreatic β-cell signalling and function. J. Mol. Endocrinol. 2016, 56, 175–187. 10.1530/jme-15-0212. 26647387

[ref24] Tampio J.; Markowicz-Piasecka M.; Huttunen K. M. Hemocompatible L-Type Amino Acid Transporter 1 (LAT1)-Utilizing Prodrugs of Perforin Inhibitors Can Accumulate into the Pancreas and Alleviate Inflammation-Induced Apoptosis. Chem.-Biol. Interact. 2021, 345, 109560 10.1016/j.cbi.2021.109560. 34153225

[ref25] Markowicz-Piasecka M.; Huttunen K. M.; Mikiciuk-Olasik E.; et al. Biocompatible Sulfenamide and Sulfonamide Derivatives of Metformin Can Exert Beneficial Effects on Plasma Haemostasis. Chem.-Biol. Interact. 2018, 280, 15–27. 10.1016/j.cbi.2017.12.005. 29217384

[ref26] Uchida Y.; Tachikawa M.; Obuchi W.; et al. A Study Protocol for Quantitative Targeted Absolute Proteomics (QTAP) by LC-MS/MS: Application for Inter-Strain Differences in Protein Expression Levels of Transporters, Receptors, Claudin-5, and Marker Proteins at the Blood-Brain Barrier in DdY, FVB, and C57BL/6J Mice. Fluids Barriers CNS 2013, 10, 21 10.1186/2045-8118-10-21. 23758935 PMC3691662

[ref27] Gynther M.; Proietti Silvestri I.; Hansen J. C.; et al. Augmentation of Anticancer Drug Efficacy in Murine Hepatocellular Carcinoma Cells by a Peripherally Acting Competitive N-Methyl-d-aspartate (NMDA) Receptor Antagonist. J. Med. Chem. 2017, 60, 9885–9904. 10.1021/acs.jmedchem.7b01624. 29205034 PMC5788303

[ref28] Montaser A. B.; Järvinen J.; Löffler S.; et al. L-Type Amino Acid Transporter 1 Enables the Efficient Brain Delivery of Small-Sized Prodrug across the Blood-Brain Barrier and into Human and Mouse Brain Parenchymal Cells. ACS Chem. Neurosci. 2020, 11, 4301–4315. 10.1021/acschemneuro.0c00564. 33228353

[ref29] Kalvass J. C.; Maurer T. S. Influence of Nonspecific Brain and Plasma Binding on CNS Exposure: Implications for Rational Drug Discovery. Biopharm. Drug Dispos. 2002, 23, 327–338. 10.1002/bdd.325. 12415573

[ref30] Tampio J.; Huttunen J.; Montaser A.; et al. Targeting of Perforin Inhibitor into the Brain Parenchyma Via a Prodrug Approach Can Decrease Oxidative Stress and Neuroinflammation and Improve Cell Survival. Mol. Neurobiol. 2020, 57, 4563–4577. 10.1007/s12035-020-02045-7. 32754897 PMC7515946

[ref31] Markowicz-Piasecka M.; Sikora J.; Mateusiak Ł.; et al. New Prodrugs of Metformin Do Not Influence the Overall Haemostasis Potential and Integrity of the Erythrocyte Membrane. Eur. J. Pharmacol. 2017, 811, 208–221. 10.1016/j.ejphar.2017.06.011. 28606852

[ref32] Markowicz-Piasecka M.; Huttunen K. M.; Sadkowska A.; et al. Pleiotropic Activity of Metformin and Its Sulfonamide Derivatives on Vascular and Platelet Haemostasis. Molecules 2019, 25, 125 10.3390/molecules25010125. PMC6982810 31905674

[ref33] Wanat K. Biological Barriers, and the Influence of Protein Binding on the Passage of Drugs across Them. Mol. Biol. Rep. 2020, 47, 3221–3231. 10.1007/s11033-020-05361-2. 32140957

[ref34] Fagerberg L.; Hallström B. M.; Oksvold P.; et al. Analysis of the Human Tissue-Specific Expression by Genome-Wide Integration of Transcriptomics and Antibody-Based Proteomics. Mol. Cell. Proteomics 2014, 13, 397–406. 10.1074/mcp.m113.035600. 24309898 PMC3916642

[ref35] Puris E.; Gynther M.; de Lange E. C. M.; et al. Mechanistic Study on the Use of the L-Type Amino Acid Transporter 1 for Brain Intracellular Delivery of Ketoprofen via Prodrug: A Novel Approach Supporting the Development of Prodrugs for Intracellular Targets. Mol. Biopharm. 2019, 16, 3261–3274. 10.1021/acs.molpharmaceut.9b00502. 31180686

[ref36] Huttunen J.; Gynther M.; Vellonen K. S.; et al. L-Type Amino Acid Transporter 1 (LAT1)-Utilizing Prodrugs Are Carrier-Selective despite Having Low Affinity for Organic Anion Transporting Polypeptides (OATPs). Int. J. Pharm. 2019, 571, 118714 10.1016/j.ijpharm.2019.118714. 31610281

[ref37] Puris E.; Gynther M.; Auriola S.; et al. L-Type Amino Acid Transporter 1 as a Target for Drug Delivery. Pharm. Res. 2020, 37, 88–104. 10.1007/s11095-020-02826-8. 32377929 PMC7203094

[ref38] Mueckler M.; Thorens B. The SLC2 (GLUT) Family of Membrane Transporters. Mol. Aspects Med. 2013, 34, 121–138. 10.1016/j.mam.2012.07.001. 23506862 PMC4104978

[ref39] Abbas A.; Beamish C.; McGirr R.; et al. Characterization of 5-(2-18F-fluoroethoxy)-L-tryptophan for PET imaging of the pancreas. F1000Research 2016, 5, 1851 10.12688/f1000research.9129.2. 27909574 PMC5112576

[ref40] Aslanoglou D.; Bertera S.; Sánchez-Soto M.; et al. Dopamine Regulates Pancreatic Glucagon and Insulin Secretion via Adrenergic and Dopaminergic Receptors. Transl. Psychiatry 2021, 11, 59 10.1038/s41398-020-01171-z. 33589583 PMC7884786

[ref41] Huttunen K. M.; Huttunen J.; Aufderhaar I.; et al. l -Type amino acid transporter 1 (lat1)-mediated targeted delivery of perforin inhibitors. Int. J. Pharm. 2016, 498, 205–216. 10.1016/j.ijpharm.2015.12.034. 26705152

[ref42] Huttunen J.; Agami M.; Tampio J.; et al. Comparison of Experimental Strategies to Study L-Type Amino Acid Transporter 1 (LAT1) Utilization by Ligands. Molecules 2021, 27, 37 10.3390/molecules27010037. 35011270 PMC8746705

[ref43] Zhao Z.; Egashira Y.; Sanada H. Ferulic Acid Sugar Esters Are Recovered in Rat Plasma and Urine Mainly as the Sulfoglucuronide of Ferulic Acid. J. Nutr. 2003, 133, 1355–1361. 10.1093/jn/133.5.1355. 12730422

[ref44] Proniewski B.; Kij A.; Sitek B.; et al. Multiorgan Development of Oxidative and Nitrosative Stress in LPS-Induced Endotoxemia in C57BL/6 Mice: DHE-Based *in Vivo* Approach. Oxid. Med. Cell. Longevity 2019, 2019, 7838406 10.1155/2019/7838406. PMC6556324 31249650

[ref45] Hamesch K.; Borkham-Kamphorst E.; Strnad P.; et al. Lipopolysaccharide-Induced Inflammatory Liver Injury in Mice. Lab. Anim. 2015, 49, 37–46. 10.1177/0023677215570087. 25835737

[ref46] Nakano D.; Kitada K.; Wan N.; et al. Lipopolysaccharide induces filtrate leakage from renal tubular lumina into the interstitial space via a proximal tubular Toll-like receptor 4-dependent pathway and limits sensitivity to fluid therapy in mice. Kidney Int. 2020, 97, 904–912. 10.1016/j.kint.2019.11.024. 32107020

[ref47] Mahapatra S.; Ying L.; Ho P. P. K.; et al. An Amyloidogenic Hexapeptide Derived from Amylin Attenuates Inflammation and Acute Lung Injury in Murine Sepsis. PLoS ONE 2018, 13, e0199206 10.1371/journal.pone.0199206. 29990318 PMC6039005

[ref48] Yanagisawa N.; Ichinoe M.; Mikami T.; et al. High Expression of L-Type Amino Acid Transporter 1 (LAT1) Predicts Poor Prognosis in Pancreatic Ductal Adenocarcinomas. J. Clin. Pathol. 2012, 65, 1019–1023. 10.1136/jclinpath-2012-200826. 22813728

[ref49] Amin K.; Dannenfelser R. M. *In vitro* hemolysis: Guidance for the pharmaceutical scientist. J. Pharm. Sci. 2006, 95, 1173–1176. 10.1002/jps.20627. 16639718

[ref50] Rudenko S. V. Erythrocyte Morphological States, Phases, Transitions and Trajectories. Biochim. Biophys. Acta, Biomembr. 2010, 1798, 1767–1778. 10.1016/j.bbamem.2010.05.010. 20538541

[ref51] Tachev K. D.; Danov K. D.; Kralchevsky P. A. On the Mechanism of Stomatocyte-Echinocyte Transformations of Red Blood Cells: Experiment and Theoretical Model. Colloids Surf., B 2004, 34, 123–140. 10.1016/j.colsurfb.2003.12.011. 15261082

