# Supplementary material for: L-type Amino Acid Transporter 1 Utilizing Ferulic Acid Derivatives Show Increased Drug Delivery in the Mouse Pancreas Along with Decreased Lipid Peroxidation and Prostaglandin Production
Source: Mol Pharm. 2022 Aug 26;19(11):3806–19. doi: 10.1021/acs.molpharmaceut.2c00328 (PMC9644403; doi:10.1021/acs.molpharmaceut.2c00328)
Supplement: Supplementary file 1 — mp2c00328_si_001.pdf [file mp2c00328_si_001.pdf]

Supporting Information

The L-type Amino Acid Transporter 1 Utilizing Ferulic Acid Derivatives Show Increased Drug Delivery in Mouse Pancreas Along with Decreased Lipid Peroxidation and Prostaglandin Production

Janne Tampio [1,], Magdalena Markowicz-Piasecka [2], Ahmed Montaser [1], Jaana Rysä [1], Anu Kauppinen [1], Kristiina M. Huttunen [1]*

[1] School of Pharmacy, Faculty of Health Sciences, University of Eastern Finland, P.O. Box 1627, FI-70211 Kuopio, Finland

[2] Laboratory of Bioanalysis, Department of Pharmaceutical Chemistry, Drug Analysis and Radiopharmacy, Medical University of Lodz, ul. Muszyńskiego 1, 90-151 Lodz, Poland

* Corresponding Author: Phone: +358405215105, E-mail: janne.tampio@uef.fi,
ORCID: 0000-0002-7526-0419

1. In vivo transporter expression with targeted quantitative proteomics method

Table S1. Peptide sequences and MRM parameters used for the protein quantification.

Protein	Gene	Peptide	Type	Retention time	Precursor ion	Product ions		
					Q1	Q3-1	Q3-2	Q3-3
LAT1	*SLC7A5*	VQDAFAAAK	St	13.3	460.75	821.40	578.33	
		VQDAFAAAK*	SIS	13.4	464.75	829.40	586.34	
4F2hc	*SLC3A2*	VAGSPGWVR	St	18	464.75	701.37	614.34	517.29
		VAGSPGWVR*	SIS	18	469.76	711.38	624.35	527.30
GLUT1	*SLC2A1*	TFDEIASGFR	St	30.8	571.78	894.43	779.40	650.36
		TFDEIASGFR*	SIS	30.8	576.78	904.44	789.42	660.37
Na^+/K^+-ATPase	*ATP1A1, ATP1A2, ATP1A3*	AAVPDAVGK	St	10.8	414.23	685.39	586.32	
		AAVPDAVGK*	SIS	10.8	418.24	693.40	594.33	

K*= Lys U-13C6; U-15N2, R*= Arg U-13C6; U-15N4, St: Standard Peptide, SIS: Stable Isotope Standard Peptide

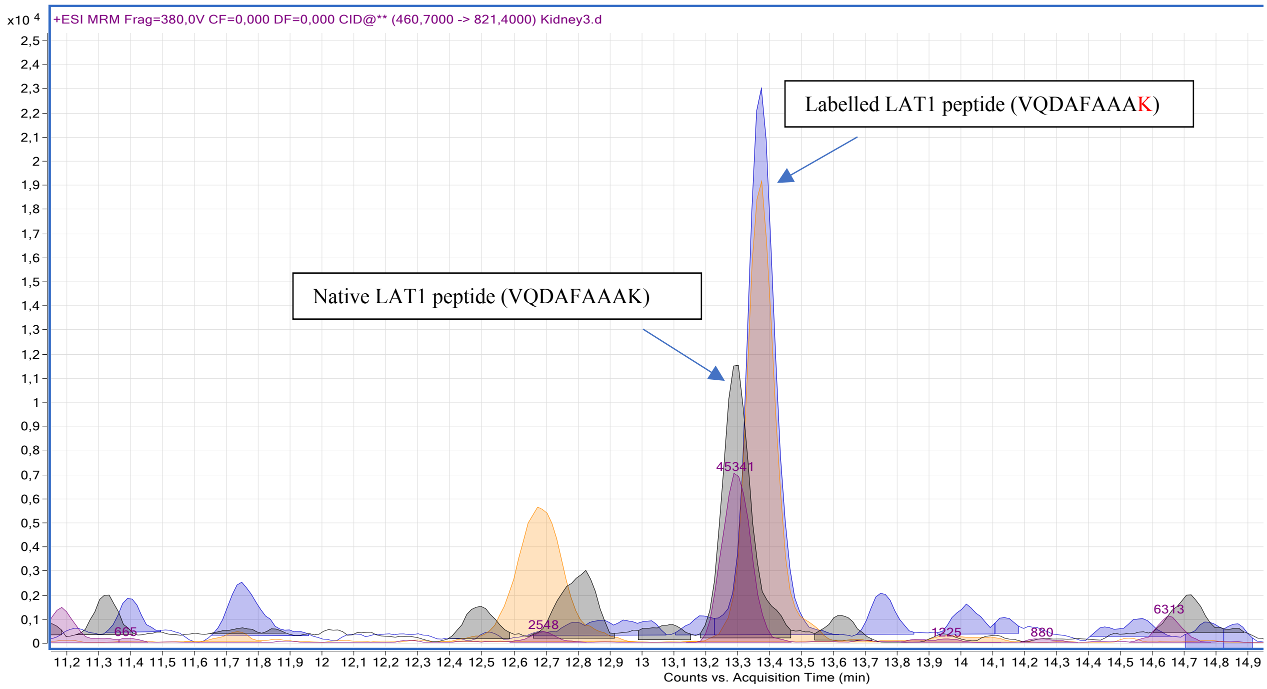

Figure S1. Example chromatograms of LAT1 peptides (VQDAFAAAK). Native and labelled peptides have been pointed.

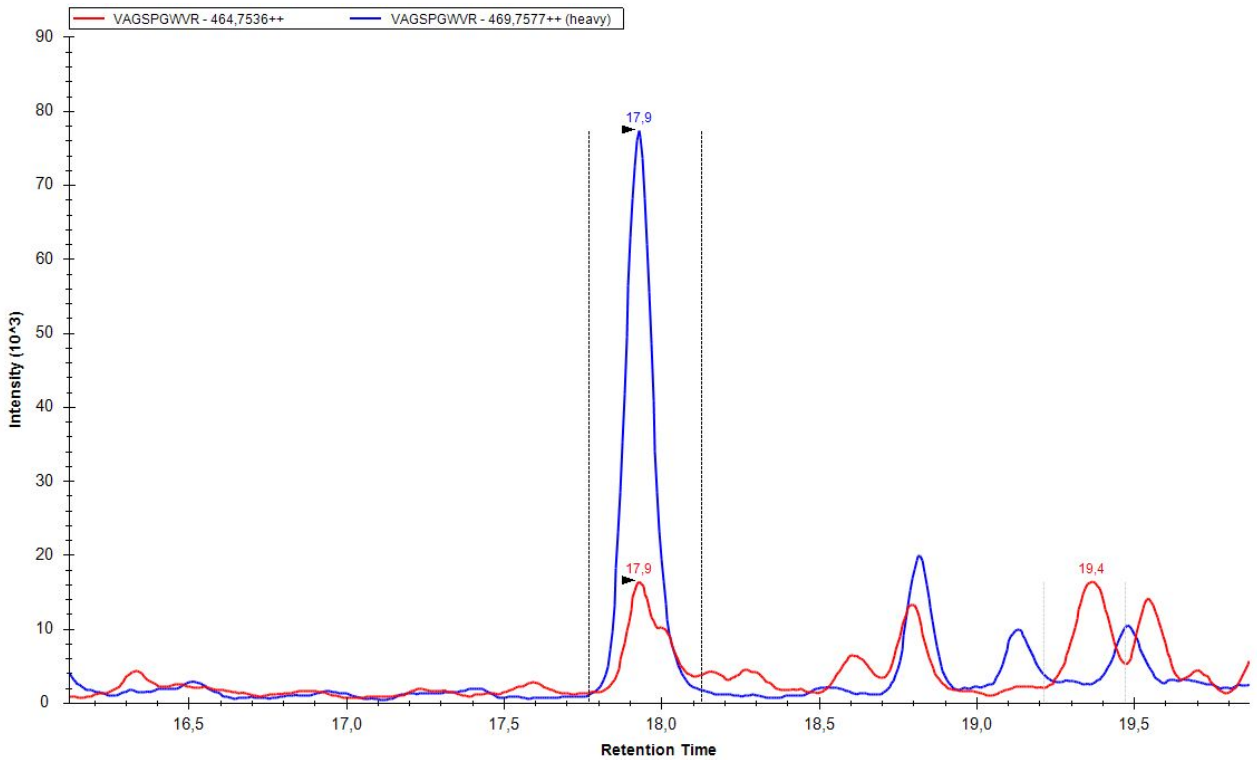

Figure S2. Example chromatograms of 4f2hc peptides. Red chromatogram is native 4f2hc peptide (VAGSPGWVR), whereas blue is an isotope labelled peptide.

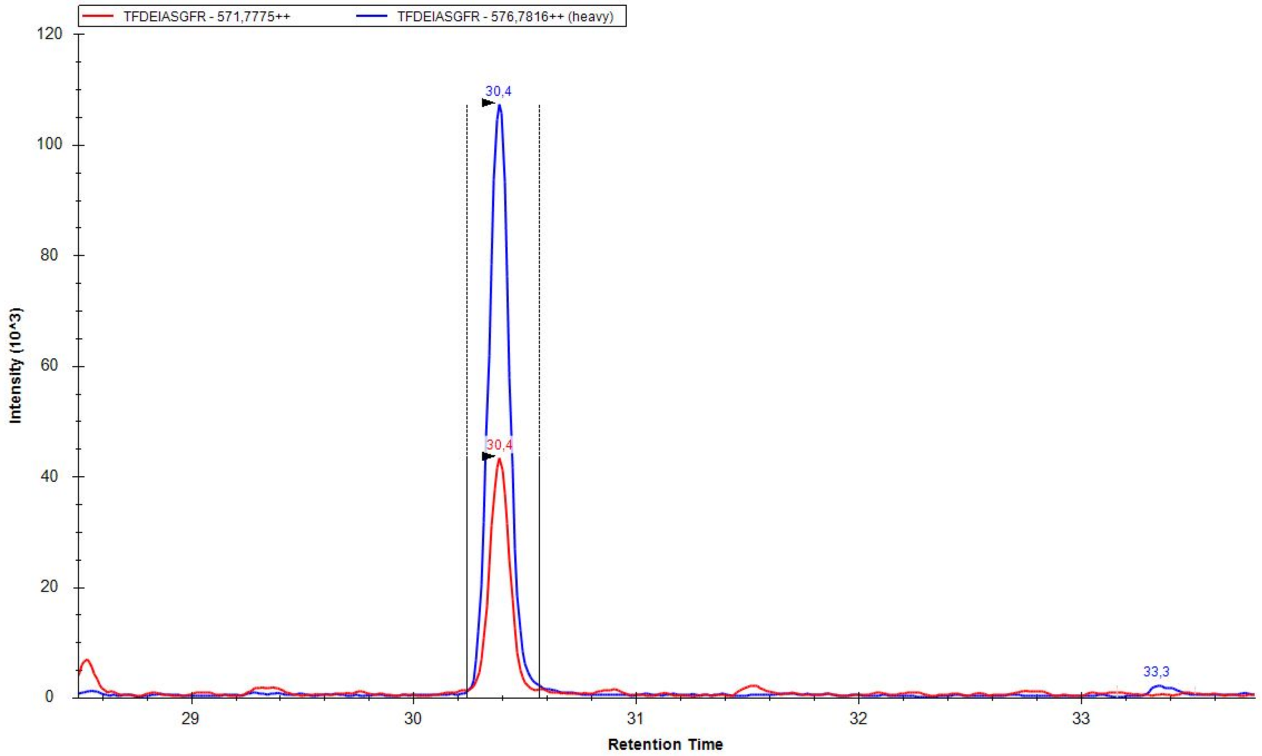

Figure S3. Example chromatograms of GLUT1 peptides. Red chromatogram is native GLUT1 peptide (TFDEIASGFR), whereas blue is an isotope labelled peptide.

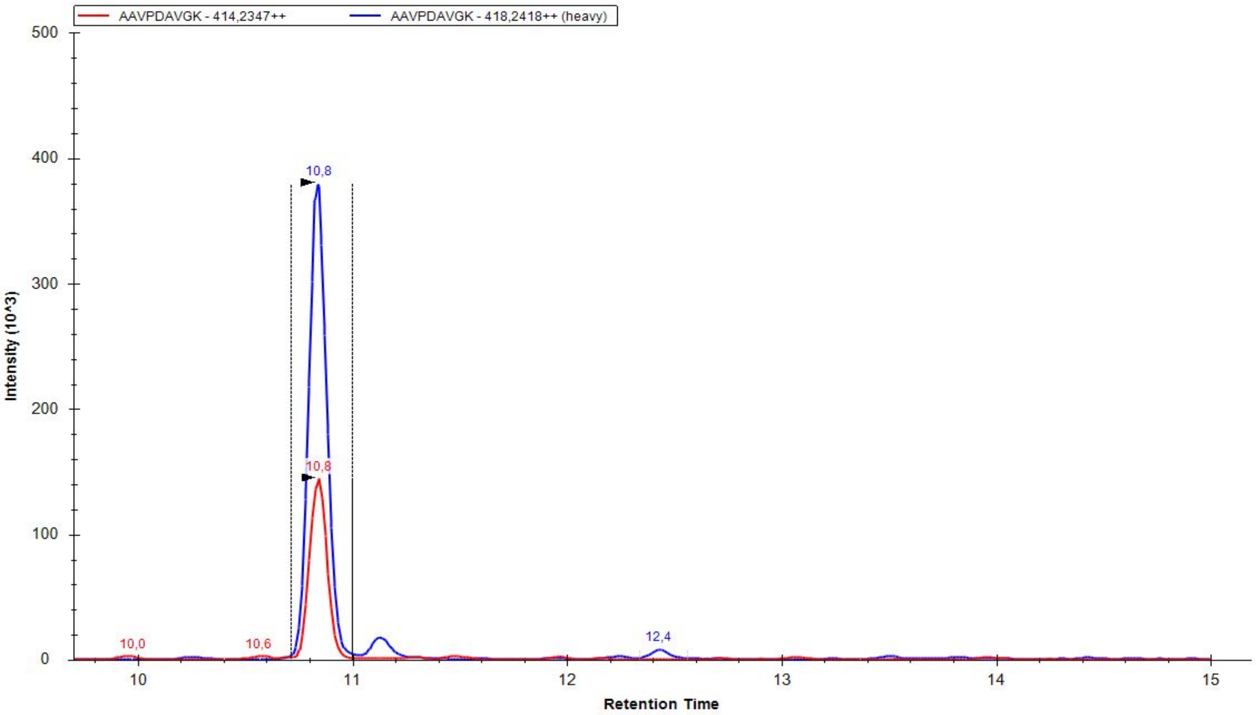

Figure S4. Example chromatograms of Na^+/K^+-ATPase peptides of the alpha subunit (1-3). Red chromatogram is native Na^+/K^+-ATPase peptide (AAVPDAVGK), whereas blue is an isotope labelled peptide.
